# Supplementary material for: Tolerogenic dendritic cell-based treatment for multiple sclerosis (MS): a harmonised study protocol for two phase I clinical trials comparing intradermal and intranodal cell administration
Source: BMJ Open. 2019 Sep 9;9(9):e030309. doi: 10.1136/bmjopen-2019-030309 (PMC6738722; doi:10.1136/bmjopen-2019-030309)
Supplement: Author's manuscript [file bmjopen-2019-030309.draft_revisions.pdf]

BMJ Open

BMJ Open is committed to open peer review. As part of this commitment we make the peer review history of every article we publish publicly available.

When an article is published we post the peer reviewers' comments and the authors' responses online. We also post the versions of the paper that were used during peer review. These are the versions that the peer review comments apply to.

The versions of the paper that follow are the versions that were submitted during the peer review process. They are not the versions of record or the final published versions. They should not be cited or distributed as the published version of this manuscript.

BMJ Open is an open access journal and the full, final, typeset and author-corrected version of record of the manuscript is available on our site with no access controls, subscription charges or pay-per-view fees (http://bmjopen.bmj.com).

If you have any questions on BMJ Open's open peer review process please email info.bmjopen@bmj.com

BMJ Open

Tolerogenic dendritic cell-based treatment for multiple sclerosis (MS): a harmonized study protocol for two phase I clinical trials comparing intradermal and intranodal cell administration

Journal:	*BMJ Open*
Manuscript ID	bmjopen-2019-030309
Article Type:	Protocol
Date Submitted by the Author:	08-Mar-2019

Complete List of Authors:	Willekens, Barbara; University Hospital Antwerp, Department of Neurology ; University of Antwerp Faculty of Medicine and Health Sciences, Laboratory of Experimental Hematology, Vaccine & Infectious Disease Institute (VAXINFECTIO)<br>Presas-Rodríguez, Silvia ; Hospital Universitari Germans Trias i Pujol, Multiple Sclerosis Unit, Department of Neurosciences; Universitat Autònoma de Barcelona, Department of Medicine<br>Mansilla, MJ; Hospital Universitario Germans Trias i Pujol and Research Institute, Division of Immunology, LCMN; Universitat Autònoma de Barcelona, Department of Cellular Biology, Physiology and Immunology<br>Derdelinckx, Judith; University Hospital Antwerp, Neurology ; University of Antwerp Faculty of Medicine and Health Sciences, Laboratory of Experimental Hematology , Vaccine & Infectious Disease Institute (VAXINFECTIO)<br>Lee, Wai-Ping; University Hospital Antwerp, Center for Cell Therapy and Regenerative Medicine<br>Nijs, Griet; University Hospital Antwerp, Center for Cell Therapy and Regenerative Medicine<br>De Laere, Maxime; University of Antwerp Faculty of Medicine and Health Sciences, Laboratory of Experimental Hematology, Vaccine & Infectious Disease Institute (VAXINFECTIO)<br>Wens, Inez; University of Antwerp Faculty of Medicine and Health Sciences, Laboratory of Experimental Hematology, Vaccine & Infectious Disease Institute (VAXINFECTIO)<br>Cras, Patrick ; University Hospital Antwerp, Department of Neurology<br>Parizel, Paul; University Hospital Antwerp, Department of Radiology; Icometrix NV<br>Van Hecke, Wim; Icometrix NV<br>Ribbens, Annemie; Icometrix NV<br>Billiet, Thibo; Icometrix NV<br>Adams, Geert; C-Clear Partners<br>Couttenye, marie-madeleine; University Hospital Antwerp, Department of Nephrology<br>Navarro-Barriuso, Juan; Hospital Universitario Germans Trias i Pujol and Research Institute, Division of Immunology, LCMN; Universitat Autònoma de Barcelona, Department of Cellular Biology, Physiology and Immunology<br>Teniente-Serra , Aina; Hospital Universitario Germans Trias i Pujol and

Research Institute, Division of Immunology, LCMN; Universitat Autònoma de Barcelona, Department of Cellular Biology, Physiology and Immunology
Quirant-Sánchez , Bibiana; Hospital Universitario Germans Trias i Pujol and Research Institute, Division of Immunology, LCMN; Universitat Autònoma de Barcelona, Department of Cellular Biology, Physiology and Immunology
Lopez-Diaz de Cerio, A; clinica universidad de navarra, Haematology-Cell Therapy Area; clinica universidad de navarra, Immunology and Immunotherapy Department
Inogés, S; clinica universidad de navarra, Haematology-Cell Therapy Area; clinica universidad de navarra, Immunology and Immunotherapy Department
Prosper, Felipe; clinica universidad de navarra, Haematology-Cell Therapy Area; Universidad de Navarra, Program of Haematology-Oncology, CIMA
Kip, Anke; Lygature
Verheij, Herman; Lygature
Gross, CC; University Hospital Munster, Department of Neurology; University of Munster, Department of Neurology
Wiendl, Heinz; University Hospital Munster, Department of Neurology; University of Munster, Department of Neurology
Van Ham, SM; Sanquin Research, Department of Immunopathology; University of Amsterdam, Landsteiner Laboratory, Academic Medical Centre
Ten Brinke, Anja; Sanquin Research, Department of Immunopathology; University of Amsterdam, Landsteiner Laboratory, Academic Medical Centre
Barriocanal, Ana Maria; Germans Trias i Pujol Health Sciences Research Institute , Clinical Research Polyvalent Unit, Clinial Trial Unit-Spanish Clinical Research Network ; Universitat Autònoma de Barcelona, Department of Pharmacology, Therapeutic and Toxicology
Massuet-Vilamajó , A; Hospital Universitari Germans Trias i Pujol, Institut de Diagnòstic per la Imatge
Hens, Niel; Universiteit Hasselt, Interuniversity Institute for Biostatistics and statistical Bioinformatics (I-BioStat); University of Antwerp Faculty of Medicine and Health Sciences, Centre for Health Economic Research and Modelling Infectious Diseases, Vaccine & Infectious Disease Institute (VAXINFECTIO)& Center for Statistics
Berneman, Zwi; University of Antwerp Faculty of Medicine and Health Sciences, Laboratory of Experimental Hematology ; University Hospital Antwerp, Center for Cell Therapy and Regenerative Medicine
Martínez-Cáceres , Eva; Hospital Universitario Germans Trias i Pujol and Research Institute, Division of Immunology, LCMN
Cools, Nathalie; University of Antwerp Faculty of Medicine and Health Sciences, Laboratory of Experimental Hematology, Vaccine & Infectious Disease Institute (VAXINFECTIO); University Hospital Antwerp, Center for Cell Therapy and Regenerative Medicine
Ramo-Tello, Cristina; Hospital Universitari Germans Trias i Pujol, Multiple Sclerosis Unit, Department of Neurosciences

Keywords: Multiple sclerosis < NEUROLOGY, Clinical trials < THERAPEUTICS, Immunology < BASIC SCIENCES, IMMUNOLOGY, Magnetic resonance imaging < RADIOLOGY & IMAGING

SCHOLARONE™
Manuscripts

Tolerogenic dendritic cell-based treatment for multiple sclerosis (MS): a harmonized study protocol for two phase I clinical trials comparing intradermal and intranodal cell administration

Willekens B[$,1,2], Presas-Rodríguez, S[$,3,4], Mansilla MJ[5,6], Derdelinckx J[1,2], Lee W-P[7], Nijs G[7], De Laere M[2], Wens I[2], Cras P[1], Parizel PM[8,9], Van Hecke W[9], Ribbens A[9], Billiet T[9], Adams G[10], Couttenye M[11], Navarro-Barriuso J[5,6], Teniente-Serra A[5,6], Quirant-Sánchez B[5,6], López-Díaz de Cerio A[12,13], Inogés S[12,13], Prósper F[12,14], Kip A[15], Verheij H[15], Gross CC[16], Wiendl H[16], van Ham SM[17], ten Brinke A[17], Barriocanal AM[18,19], Massuet-Vilamajó A[20], Hens N[21], Berneman Z[2,7], Martínez-Cáceres EM[5], Cools N[$,2,7*] and Ramo-Tello C[$,3] on behalf of the RESTORE consortium

[$]these authors contributed equally to this paper

*corresponding author: Nathalie.Cools@uza.be

Author affiliations:

1 Department of Neurology, Antwerp University Hospital, Edegem, Belgium

2 Laboratory of Experimental Hematology, Vaccine & Infectious Disease Institute (VAXINFECTIO), Faculty of Medicine and Health Sciences, University of Antwerp, Wilrijk, Belgium

3 Multiple Sclerosis Unit, Department of Neurosciences, Hospital Universitari Germans Trias i Pujol, Badalona, Spain

4 Department of Medicine, Universitat Autònoma de Barcelona, Cerdanyola del Vallés, Spain

5 Division of Immunology,LCMN, Hospital Universitario Germans Trias i Pujol and Research Institute, Campus Can Ruti, Badalona, Spain

For peer review only - http://bmjopen.bmj.com/site/about/guidelines.xhtml

Study protocol for tolDC administration in patients with active MS

6 Department of Cellular Biology, Physiology and Immunology, Universitat Autònoma de Barcelona, Bellaterra, Spain

7 Center for Cell Therapy and Regenerative Medicine, Antwerp University Hospital, Edegem, Belgium

8 Department of Radiology, Antwerp University Hospital, Edegem, Belgium

9 Icometrix, Kolonel Begaultlaan 1b, Leuven, Belgium

10 C-Clear Partners, Mortsel, Belgium

11 Department of Nephrology, Antwerp University Hospital, Edegem, Belgium

12 Haematology-Cell Therapy Area, Clinica Universidad de Navarra, Pamplona, Spain

13 Immunology and Immunotherapy Department, Clinica Universidad de Navarra, Pamplona, Spain

14 Program of Haematology-Oncology, CIMA, Universidad de Navarra, Pamplona, Spain

15 Lygature, Utrecht, The Netherlands

16 Department of Neurology, University Hospital and University Münster, Münster, Germany

17 Department of Immunopathology, Sanquin Research, Amsterdam, The Netherlands and Landsteiner Laboratory, Academic Medical Centre, University of Amsterdam, Amsterdam, The Netherlands

18 Clinical Research Polyvalent Unit, Germans Trias i Pujol Health Sciences Research Institute (IGTP), Badalona, Spain. Clinial Trial Unit-Spanish Clinical Research Network SCReN.

19 Department of Pharmacology, Therapeutic and Toxicology, Universitat Autònoma de Barcelona, Cerdanyola del Vallès, Spain

20 Institut de Diagnòstic per la Imatge, Hospital Universitari Germans Trias i Pujol, Badalona, Spain

21 Centre for Health Economic Research and Modelling Infectious Diseases, Vaccine & Infectious Disease Institute (VAXINFECTIO), Wilrijk, Belgium & Center for Statistics, I-BioStat, Hasselt University, Hasselt, Belgium

For peer review only

ABSTRACT

Introduction: Based on the extraordinary advances in the treatment of multiple sclerosis (MS), currently available disease-modifying treatments (DMT) have positively influenced the disease course of MS. However, higher treatment efficacy comes with a more severe risk profile. Hence, the unmet need for safer and more selective treatments remains. Specifically restoring immune tolerance towards myelin antigens may provide an attractive alternative. In this respect, antigen-specific tolerization with autologous tolerogenic dendritic cells (tolDC) is a promising approach.

Methods and analysis: Here, we will evaluate the clinical use of tolDC in a well-defined population of MS patients in two phase I clinical trials. In doing so, we aim to compare two ways of tolDC administration, namely intradermal and intranodal. The cells will be injected at consecutive intervals in 3 cohorts receiving incremental doses of tolDC, according to a best-of-five design. The primary objective is to assess the safety and feasibility of tolDC administration. For safety, the number of adverse events (AE) including magnetic resonance imaging (MRI) and clinical outcomes will be assessed. For feasibility, successful production of tolDC will be determined. Secondary endpoints include clinical and MRI outcome measures. The patients' immune profile will be assessed to find presumptive evidence for a tolerogenic effect *in vivo*.

Ethics and dissemination: Ethics approval was obtained for the two phase I clinical trials. The results of the trials will be disseminated in a peer-reviewed journal, at scientific conferences and to patient associations.

Trial registration numbers: Both studies are registered at clinicaltrials.gov: NCT02618902 and NCT02903537; EudraCT numbers are 2015-002975-16 and 2015-003541-26, respectively.

For peer review only

STRENGTHS AND LIMITATIONS OF THIS STUDY

- The concept of the clinical trials is built on recent advances in the understanding of tolerance induction via cell-based therapy, and the effort made to precisely silence myelin-antigen-specific, putatively deleterious immune responses in the disease.
- The use of cryopreserved tolDC allows producing batches of tolDC, stored in ready-to-use aliquots for the required administrations during the treatment period, thereby reducing variability and global production cost.
- Harmonization of clinical, MRI and immunological evaluations of the patients will enable us to compare results between two phase I clinical trials evaluating the safety and feasibility of autologous tolDC administration in patients with active MS.
- The patients are not randomized across the trials, limiting direct comparison of both routes of administration.
- The assessors are not blinded in the phase I clinical trials.

For peer review only

INTRODUCTION

Multiple sclerosis (MS) is the leading cause of non-traumatic disability in young adults and typically presents between the age of 20-40 years, in the prime of a patient's personal and professional life. The heterogeneity of the disease course in MS, i.e. relapsing-remitting or progressive, remains a challenge for patient management and design of clinical trials. According to Lublin et al.[1], MS is categorized as relapsing or progressive. In both forms, disease activity (defined by clinical relapse and/or MRI lesions) and disease progression are measured. To date, more than 10 disease-modifying treatments (DMT) are approved for clinical use in patients with active relapsing-remitting MS (RRMS). This has dramatically influenced the management and prognosis of MS. Indeed, whereas earlier epidemiological studies indicated that 50% of patients develop secondary progressive MS (SPMS) after 15 years when untreated[2], only 18% of patients treated with modern MS therapeutics were reported to evolve to a secondary progressive course after a median duration of 16.8 years in a recent cohort[3]. Nonetheless, DMT are not specific nor selective for MS, i.e. they work in an immunomodulatory or immunosuppressive way by sequestering or depleting lymphocytes, albeit that some therapies can induce immune reconstitution as well. Moreover, treatment-related side effects or risks can be severe[4], leaving a significant and unmet need for safer and more disease-selective treatments.

The crux of MS is the patient's own immune cells attacking self-antigens in the central nervous system (CNS), caused by a loss of tolerance against myelin antigens. This is manifested by inflammatory infiltrates, demyelination and axonal loss, resulting in the clinical symptoms of the disease[5 6]. Previously, it was shown that proteins expressed in the myelin sheath, protecting neuronal axons of the CNS, are an important target of the autoreactive T cell response[7-9]. Also in our hands, *ex vivo* T cell reactivity against a mix of 7

For peer review only

immunodominant myelin-derived peptides could be demonstrated in RRMS patients as compared to healthy controls and patients with other neurological disorders[10]. Although the underlying cause of the loss of tolerance towards myelin antigens has not been elucidated yet, one ultimate aim in the treatment of MS is to re-establish antigen-specific immune tolerance towards CNS structures[11-15]. In this perspective, it is postulated that tolerance-inducing antigen-specific therapy can be an innovative and promising strategy for the treatment of autoimmunity. This approach is based on the possibility that autoreactive B and T cells, driving myelin destruction and damage in the CNS, will be eradicated by inducing tolerance to myelin-derived peptides, without interfering with protective immunity. Several approaches involving the induction of antigen-specific tolerance have reached the clinical development phase and demonstrated promising results in phase I/II clinical trials (extensively reviewed by Willekens and Cools[12]). Given the fact that dendritic cells (DC) play a key role in controlling the immune response by steering the outcome of antigen presentation to T cells, the use of tolerance-inducing or tolerogenic dendritic cells (tolDC) may provide prospect for the treatment of MS[16-20]. Recently, we demonstrated the potential of 1α,25-dihydroxyvitamin D_3 to generate tolDC from MS patients[21-23]. Indeed, vitamin D_3-treated tolDC from MS patients display a maturation-resistant phenotype, i.e. they maintain their low expression levels of costimulatory molecules (CD80, CD83, CD86) and anti-inflammatory cytokine profile, even following re-challenge with an inflammatory stimulus such as lipopolysaccharide (LPS). Furthermore, tolDC from MS patients are capable of inducing stable and antigen-specific T cell hyporesponsiveness. After *in vitro* stimulation with myelin-derived peptide-pulsed vitamin D_3-treated tolDC, T cells were unresponsive to the myelin peptides used, while retaining their capacity to respond to an unrelated antigen.

Moreover, T cell hyporesponsiveness was robust, as T cells were not reactivated after rechallenge with immunostimulatory DC[21-23].

Also *in vivo*, the administration of bone marrow-derived vitamin D_3-treated tolDC pulsed with MOG_{40-55} has been shown to induce antigen-specific T cell tolerance in experimental autoimmune encephalomyelitis (EAE), the animal model of MS. Mice treated with MOG_{40-55}-pulsed bone marrow-derived vitamin D_3-treated tolDC before disease induction showed reduced incidence of the disease. Furthermore, when the treatment was used therapeutically in mice already showing clinical signs of the disease, the severity of the disease was significantly reduced[24]. However, repetitive injections of tolDC were necessary for a prolonged clinical effect in EAE. In this context, cryopreserved MOG_{40-55}-pulsed tolDC demonstrated similar clinical benefit compared to fresh tolDC, underlining the therapeutic potential and clinical applicability of cryopreserved tolDC[21 25].

Recently, a number of phase I studies investigating the safety and feasibility of tolDC therapy for autoimmune diseases such as rheumatic arthritis, type I diabetes, or Crohn's disease were completed[18]. The first results are highly encouraging since none of the trials found safety concerns related to tolDC administration in these patients. TolDC were well tolerated and autoimmunity was not exacerbated in the patients treated[26-29]. Nevertheless, numerous questions remain, and the efficacy of antigen-specific tolDC therapy may depend on many factors, of which the route of administration is among the most important. When considering the route of delivery of DC, one needs to take into account that different routes lead to different sites of accumulation of the vaccinated DC. In most clinical studies to date, *ex vivo* generated DC were injected either intravenously, intradermally or subcutaneously. In humans, migration of DC towards the secondary lymph nodes is superior after intradermal injection compared with after subcutaneous injection of DC[30-32], whereas migration of

For peer review only - http://bmjopen.bmj.com/site/about/guidelines.xhtml

intravenously injected DC has not been monitored so far. Direct delivery of DC to lymph nodes via intranodal injection showed promising results in therapies using immunostimulatory DC in cancer[33 34], but has not been evaluated for tolDC. Until now, there are no available data showing superiority of one route over others for the administration of peptide-loaded tolDC. Here, we will compare intradermal (i.d.) injection to intranodal (i.n.) injection. Only the direct comparison of these different routes of administration in two dose-escalation studies will allow us to determine if both routes are equally safe.

In conclusion, our objectives are to evaluate safety, clinical feasibility and the immunological consequences of peptide-loaded tolDC administered intranodal or intradermal in MS patients in two clinical trials. Harmonization of the procedures for clinical, MRI as well as immune-monitoring will enable us to compare results between trials of which the study protocols will be discussed in detail here.

For peer review only

METHODS AND ANALYSIS

Study design

Two open-label, dose-escalation phase I clinical trials, MS-tolDC and TOLERVIT-MS, are designed in a coordinated and comprehensive manner and run simultaneously in Belgium (Antwerp University Hospital, Edegem) and Spain (Hospital Germans Trias i Pujol, Badalona), respectively. Ethics approval was obtained for both phase I clinical trials. Patient recruitment started mid-2017 and is anticipated to end in 2019.

Primary objectives

To evaluate the safety of administering tolDC, the occurrence and severity of adverse events (AE) will be recorded. To assess feasibility, successful production of tolDC according to good manufacturing practices (GMP) starting from a leukapheresis procedure will be assessed.

Secondary objectives

Preliminary efficacy measures, including clinical outcomes and brain MRI, will be evaluated. In addition, whole blood lymphocyte phenotyping and cytokine profiling will be assessed before and after completion of the vaccination cycle, as well as the ability of tolDC to suppress pathogenic T cell responses. For this, myelin-specific T cell reactivity will be determined before and after completion of the vaccination cycle. All secondary objectives contribute to determination of proof of principle.

Patient-reported outcomes

MS QOL-54 will be evaluated to detect changes in general and disease-specific quality of life.

For peer review only

Study population

Eligibility and enrolment

Patients with active relapsing-remitting and active progressive MS, diagnosed according to most recent McDonald criteria[35 36], and who are not eligible for or do not want to be treated with currently available DMT, will be recruited. Patients are included after written informed consent and enrolled in the study when the in- and exclusion criteria are met (Table 1).

Determination of sample size and dose-escalation procedure

The studies will be conducted according to a "best of five" design[37], an alternative of the traditional 3+3 design in that one additional patient is added when one or even two dose-limiting toxicities (DLT) are observed among the first three patients. Another patient is added when two DLT are observed among four treated patients. Dose escalation is allowed if DLT are observed among none of three, one of four, or two of five patients, but the trial will terminate if three or more DLT are observed. A DLT is defined as a serious adverse event (SAE) that is attributable to the study cells administered, or of which the severity prevents further escalation. Dose escalation decisions will be made after all subjects in the cohort have completed at least 3 months of follow up and when the results of the safety and tolerability analyses of the preceding dose regimen are satisfactory in the judgment of the investigators and the independent Data Safety Monitoring Board (DSMB).

An overview of the dose escalation is provided in Table 2. Altogether, each phase I study is intended to accrue a total number of 9-15 evaluable patients.

Study medication

Generation of tolDC will be carried out according to the principles and guidelines of GMP laid down in Directive 2003/94/EC. TolDC production will be performed in the GMP facilities of

For peer review only - http://bmjopen.bmj.com/site/about/guidelines.xhtml

the Center for Cell Therapy and Regenerative Medicine (CCRG) of the Antwerp University Hospital (Belgium) and of the Cell Therapy Area of the Clínica Universidad de Navarra (Spain).

Clinical-grade autologous tolDC will be prepared from a leukapheresis. $CD14^+$ monocytes will be cultured in GMP-grade cell culture medium supplemented with 2% human AB serum, granulocyte macrophage colony-stimulating factor (GM-CSF), interleukin (IL)-4 and 1α,25 dihydroxyvitamin D_3. At day 4, tolDC will be stimulated using an inflammatory cytokine cocktail, consisting of tumor necrosis factor (TNF)-α, prostaglandin (PG) E_2 and IL-1β. At day 6, tolDC will be harvested, loaded with 7 myelin antigens (MBP_{13-32}, $MBP_{111-129}$, $MBP_{154-170}$, $PLP_{139-154}$, MOG_{1-20}, MOG_{35-55} and MBP_{83-99}), and cryopreserved at -196°C. Separate aliquots of the cell product will be prepared for quality control and quality assurance (QC/QA). This includes (i) sterility testing, (ii) cell count, (iii) viability, (iv) flow cytometric phenotyping, and (v) induction of T cell hyporesponsiveness in an allogeneic mixed leukocyte reaction (allo-MLR).

Trial intervention: tolDC administration

In this study, 6 vaccine doses will be administered to each participant according to the following immunization schedule: a total of 6 vaccine doses (V), with V_{1-4} at biweekly (± 3 days) intervals and V_{5-6} at monthly intervals (± 3 days). A complete overview of the study design is depicted in Figure 1. Detailed assessments per visit are shown in Table 3.

In Belgium (MS-tolDC), tolDC vaccination occurs through intradermal injection at 5 alternating sites (100µL/site) in the posterior neck region to ensure lymphatic drainage to superficial and deep cervical lymph nodes (5-10 cm from the cervical lymph nodes). In addition, in Spain (TOLERVIT-MS), tolDC will be injected directly in the cervical lymph nodes under echographic guidance by an expert physician. If the patient's anatomical conditions do

For peer review only

not allow it (e.g., small lymph nodes), the vaccine will be distributed in more nodes (max500µL/node).

For peer review only

For peer review only - http://bmjopen.bmj.com/site/about/guidelines.xhtml

Primary outcome measures

Safety

To evaluate the safety of administered tolDC, occurrence of AE will be recorded using clinical outcome measures, i.e. physical (skin, pain, adenopathies) and neurological examination (relapses, worsening disability) and non-clinical outcome measures, i.e. brain MRI, biochemical and hematological safety. The frequent MRI monitoring will allow us to monitor the safety of tolDC in MS patients objectively by measuring T1-enhancing lesions and new and/or enlarging T2 lesions. In this way, unexpected disease activity can be detected timely. The severity of AE will be defined according to the World Health Organization (WHO) toxicity grading scale. The relationship of an AE to the investigational product will be determined by the neurologists and the independent DSMB on the basis of their clinical judgment.

A proportion of the patients may experience MS relapses during the study. A relapse is defined as a new or worsening neurological symptom that occurs in the absence of fever or infection, occurs at least 30 days following the onset of a previous relapse, persists for at least 24 hours and includes an increase in EDSS (Expanded Disability Status Scale) score[38] from the previous assessment matching one of the following: (i) an increase of ≥ 1 on the total scale score; or (ii) an increase of ≥ 2 points on one of the appropriate Functional System Scores (FSS), or (iii) an increase of > 1 point on two or more of the appropriate FSS. Patients will be instructed to notify their neurologist as soon as possible but within 72 hours the latest and will be examined within 7 days of onset of the symptoms. In case of a MS relapse, a course of high-dose steroids can be administered at the discretion of the treating neurologist.

For peer review only - http://bmjopen.bmj.com/site/about/guidelines.xhtml

Study treatment must be discontinued for a given patient if the investigator determines that continuing would result in a significant risk for that patient. In case of terminating the study treatment, patients will still be monitored for safety issues for the duration of the study.

Feasibility

To evaluate the feasibility, successful production of tolDC after leukapheresis will be evaluated. This includes the production of sufficient numbers of tolDC and compliance of the final investigational product to the pre-specified release criteria following cryopreservation.

Secondary outcome measures

Clinical evaluation

The following clinical and laboratory assessments will be performed during a complete physical examination: vital signs, electrocardiogram, routine blood and urine samples, urine pregnancy test in female patients of reproductive potential and serological screening tests, according to the study schedule (Figure 1 and Table 3). Concurrent drug use or new adverse events will be reviewed and recorded.

EDSS is based on a standardized neurological examination and measures impairments in 8 functional systems, including vision, brainstem, pyramidal, cerebellar, sensory, bowel and bladder, mental (cerebral), and ambulation . It has been used for over 20 years as a clinical outcome measure of MS disease progression and consists of a 10-point scale of disease severity ranging from 0, i.e. no disability, to 10, i.e. death from MS[38]. All neurologists involved in patient evaluation will be EDSS training certified.

EDSS will be supplemented by three well-known, quantitative, continuous tests that evaluate ambulation (walking speed by the timed 25 foot walk test or T25FW, arm dexterity

For peer review only - http://bmjopen.bmj.com/site/about/guidelines.xhtml

and function by the 9 Hole Peg Test or 9HPT, and cognition by the Symbol Digit Modalities Test or SDMT).

The effect on disability progression will be characterized by reporting the proportion of patients who are free from disease progression. For this, disability will be assessed on the basis of a sustained clinically relevant change seen in any one of disability assessments: EDSS, T25FW, SDMT or the 9-HTP. Disease progression is defined by: 3-month sustained increase from baseline in the EDSS score (1 point in patients with baseline EDSS score 0 to 5.0; 0.5 point in patients with baseline EDSS score of 5.5 -6.5) or 3-month sustained increase of at least 20% from baseline in the time taken to complete the T25FW or 3-month sustained increase of at least 20% from baseline in the time taken to complete the 9-HPT. Percent change from baseline after treatment in SDMT score will also be recorded.

MRI acquisition and evaluation

MRI is the current gold standard for non-clinical monitoring of MS, and MRI-derived markers have been established as standard outcome measures to monitor the treatment response in various MS clinical trials. MRI will be performed on the same 3T scanner throughout the study. The MRI protocol includes a 3PLANE scout, 3D T1-weighted image pre- and post-gadolinium (Dotarem®, 20 ml) administration (voxel resolution 0.9x0.9x0.9, TR2300.0, TE2.29, TI 900.0), and a 3D FLAIR image (voxel resolution 0.4x0.4x0.9, TR5000.0, TE387.0, TI1800.0). The quantification of number of T2 lesions, T2 lesion load, number and volume of Gd-enhancing lesions, based on 3D FLAIR images and 3D T1-weighted images, is completely automatic avoiding inter- or intra-rater variability. The lesions are quantified within different brain regions. Disease activity or progression on MRI will be evaluated by pre-treatment versus ongoing and post-treatment: (i) change in mean number of enhancing lesions; (ii) change in number of T1 Gd-enhancing and/or new or enlarging T2 lesions; (iii) percent

For peer review only - http://bmjopen.bmj.com/site/about/guidelines.xhtml

change in T2 lesion load; (iv) percent change in brain volume. In order to compare MRI outcome measures from different studies, a standardized acquisition is guaranteed by (i) a uniform MRI protocol, (ii) the use of standard operating procedures for image acquisition and upload, and (iii) training of local MRI operators and study coordinators for acquisition and upload.

Immune-monitoring

To evaluate therapy-related changes in the immune cell profile, peripheral blood (10x10 mL heparin tubes) will be sampled according to the time points depicted in Figure 1 and Table 3, and analyzed by multi-parameter flow cytometry prior to, during and after vaccination. In an attempt to cover the main leukocyte subsets of peripheral blood, the following subsets and their activation status will be enumerated in whole blood samples: $CD4^+$ and $CD8^+$ T cell subpopulations, B cell subsets, natural killer (NK) cells, NKT cells, and myeloid cells. Myelin-specific T cell reactivity will be determined before, during and after completion of administration of tolDC. For this, responsiveness of T cells to myelin antigens will be investigated. As a control, the responsiveness of T cells to unrelated antigens, e.g. cytomegalovirus (CMV) or tetanus toxoid (TT), will be addressed. In doing so, we will be able to assess *ex vivo* the potential risk of inducing opportunistic infections by administering tolDC to MS patients. Finally, patient materials from the studies will be specifically biobanked. We envisage to maximally perform batch measurements and centralized immune monitoring analysis as soon as a dose cohort has reached 3 months follow-up of all patients. For this, extensive immune cell profiling as well as cytokine production of T cells will be analyzed by multi-parameter flow cytometry. Using lineage-specific as well as activation markers, proportions and activation status of the different immune cell subsets will be determined. Besides, antibody titers and memory B cell analysis will be performed.

For peer review only - http://bmjopen.bmj.com/site/about/guidelines.xhtml

Patient-reported outcomes

Patient-reported outcomes will be assessed by Multiple Sclerosis Quality of Life-54 (MSQOL-54)[39], a self-reporting questionnaire to detect changes in general and disease-specific quality of life.

Data management and monitoring

Adequate and accurate patient records will be kept enabling the appropriate and required documentation of the study and subsequent verification of the collected data. All data are completed in the electronic case report form (eCRF) for each patient enrolled in this study, including patients who did not start with the investigational treatment. Source data verification will be performed by a central data manager affiliated to a clinical research organization (CRO). In doing so, study compliance will be monitored, thereby assuring the protection of the rights, safety and well-being of study subjects.

Analysis

Given the design of the study (phase I) and its specific primary end point (safety), no confirmatory statistical testing will be performed. P-values will be calculated but interpreted with caution. Study investigations will be exploratory and conclusions will be based on the complete set of patient evidence.

Analyses of safety variables will be performed with the safety population (all the patients treated with at least one dose of the cell product) by available data only (ADO) analysis. For demographic and safety analysis, i.e. the incidence of adverse events, laboratory values and changes in vital signs, descriptive statistics will be calculated including frequencies for categorical variables and mean and standard deviation, or median and interquartile range for quantitative parameters. In brief, EDSS and other repeatedly measured variables will be

For peer review only - http://bmjopen.bmj.com/site/about/guidelines.xhtml

analyzed by means of Mixed Models for Repeated Measurements (MMRM). In case model assumptions are violated (e.g. non-Gaussian errors) or when dealing with ordinal efficacy variables, alternative methods (including non-parametric methods and ordinal regression methods for repeated measurements) will be used. The percentage of patients with 1-point in EDSS improvement will be estimated using a binomial regression model including the treatment and the baseline EDSS. The remainder of variables will be analyzed according to the appropriate statistical test: Chi-square or Fisher's exact test to compare categorical variables, the dependent or independent t-test for continuous Gaussian-distributed variables and the Wilcoxon or Mann-Whitney test for ordinal and non-Gaussian continuous data. The significance level alpha will be set at 0.05 for two-tailed analysis. Variables to assess the analysis of secondary and tertiary outcome measures will be performed with per protocol population. Continuous variables as a minimum will be described by number of total and non-missing observations (n) and the appropriate location-scale statistics including arithmetic mean, standard deviation (SD), minimum, median, Q1-Q3, and maximum. Categorical variables will be presented using the number of non-missing observations (n) or the number of patients in the population (N) as applicable and percentages (%). Two-sided 95% (exact) Confidence Intervals (CI) will be provided when relevant.

For peer review only

Ethics and dissemination

Institutional Review Board approval

The study protocol was approved by the Federal Agency for Medicines and Health Products (FAMHP, Belgium), the Spanish Agency of Medicines and Medical Devices (Spain), the Ethics Committee of the Antwerp University Hospital (Belgium), and the Ethics Committees of Hospital Germans Trias i Pujol and Clínica Universidad de Navarra.

For peer review only - http://bmjopen.bmj.com/site/about/guidelines.xhtml

Patient protection

The study will be conducted in agreement with either the Declaration of Helsinki or the laws and regulations of the country, whichever provides the greatest protection of the patient. In the Belgian context, the Law of May 7, 2004 ("Wet van 7 mei 2004 inzake experimenten op de menselijke persoon") applies. The study will be conducted in agreement with the ICH Harmonized Tripartite Guideline for Good Clinical Practice.

Informed consent

All patients will be informed of the aim of the study, the possible adverse events, the procedures and possible hazards to which he/she will be exposed. They will be informed as to the strict confidentiality of their patient data, but that their medical records may be reviewed for trial purposes by authorized individuals other than their treating physician.

For peer review only

Dissemination

The results of the clinical trials will be published in a peer-reviewed journal. In addition, the results will be presented at scientific conferences and to patient associations. In particular for Spanish patient associations, dissemination of results will be handled by the Spanish Agency of Medicines and Medical Devices whose content is written in layman's terms. On completion of the trial and after publication of the study results, data requests can be submitted to the researchers.

For peer review only

For peer review only - http://bmjopen.bmj.com/site/about/guidelines.xhtml

DISCUSSION

Although the first phase I clinical trials have demonstrated promising results with regard to safety of administering tolDC in other autoimmune diseases, numerous questions remain concerning which dose, treatment schedule or route of administration is best with regard to safety, efficacy and related costs of treatment with tolDC. In this collaborative effort, each patient will receive 6 repetitive injections of 5, 10 or 15 x 10^6 autologous myelin-derived peptide mix-loaded tolDC, intradermal or intranodal, i.e. 4 administrations once every 2 weeks and 2 administrations once every 4 weeks. Previously, others evaluated the safety of intravenous administration of tolerogenic DC in MS and neuromyelitis optica (NMO) patients[40]. In the current study, harmonization of clinical, MRI and immunological evaluations of the patients will enable us to compare results between two phase I clinical trials evaluating the safety and feasibility of autologous tolDC administration in patients with active MS. To our knowledge, this will be the first time that different routes of administration are set side by side for a cell therapy product.

In conclusion, our protocols envisage to restore tolerance to predefined myelin-peptide antigens using peptide-loaded tolDC. From the results of these two phase I clinical studies, the optimal dose and administration route will be selected for future phase II trials investigating the efficacy of this patient-tailored treatment in MS.

For peer review only - http://bmjopen.bmj.com/site/about/guidelines.xhtml

Acknowledgements

We thank all the patients that contributed to the preclinical research by donating samples. Our gratitude goes to Marleen Breuls, MS nurse and to the study nurses of the Neurology Department of the Antwerp University Hospital, Linda Wagemaekers, Caroline Vinck and Maren Wyckmans.

For peer review only

For peer review only - http://bmjopen.bmj.com/site/about/guidelines.xhtml

Author contributions

BW, ZB, CRT, EMC, WVH and NC conceived and designed the study. WPL, GN, MDL, GA, AR, MC, IW and NC participated in logistical planning of the study. BW and SPR wrote the initial draft of the manuscript. BW, SPR, CRT, MJM, IW, EMC and NC wrote and reviewed the manuscript. NH provided the statistical support for the sample size estimates and the design of the statistical analysis. All authors made significant contributions to the development and conceptualization of the protocol. All authors reviewed the draft versions of this paper and have read and approved the final manuscript.

For peer review only

Funding

This work was supported by positive discussion through the A FACTT network (Cost Action BM1305: www.afactt.eu). COST is supported by the EU Framework Program Horizon 2020. This RESTORE project has received funding from the European Union's Horizon 2020 research and innovation program under grant agreement number 779316. Further support was provided by an applied biomedical research project of the Institute for the Promotion of Innovation by Science and Technology in Flanders (IWT-TBM 140191), by projects PI11/02416, PI14/01175,PI16/01737 and PT13/0002/0038 (Platform for Clinical Research and Clinical Trial Units, Spanish Clinical Research Network, SCReN), integrated in the Plan Nacional de I+D+I and co-supported by the Health Institute Carlos III - Subdirección General de Evaluación y Fomento de la Investigación of the Spanish Ministry of Economy and Competitiveness and the Fondo Europeo de Desarrollo Regional (FEDER), and project 07/2410 Fundació La Marato de TV3. Furthermore, the authors received research funding from Sanofi Genzyme, Belgium. Judith Derdelinckx holds a PhD fellowship from the Research Foundation Flanders (FWO). Dr. Presas-Rodríguez is a neurologist who has received a grant of Hospital Germans Trias i Pujol ("Germans Trias Talents 2016-2018") to work on this project. Dr. Willekens is a neurologist at the Antwerp University Hospital supported by a research fellowship (2016-2018) of the University of Antwerp to work on this project and she currently holds a clinical PhD fellowship from the Research Foundation Flanders (FWO 1701919N)

For peer review only - http://bmjopen.bmj.com/site/about/guidelines.xhtml

Conflict of interest

CCG received speaker honoraria and travel expenses for attending meeting from Genzyme, Novartis Pharma GmbH, and Bayer Health Care. Her work is funded by the German Ministry for Education and Research (BMBF; 01GI1603A) and the German Research Foundation (DFG; GR3946/3-1 and SFB128 A09).

HW receives honoraria for acting as a member of Scientific Advisory Boards and as consultant for Biogen, Evgen, MedDay Pharmaceuticals, Merck Serono, Novartis, Roche Pharma AG, Sanofi-Genzyme, as well as speaker honoraria and travel support from Alexion, Biogen, Cognomed, F. Hoffmann-La Roche Ltd., Gemeinnützige Hertie-Stiftung, Merck Serono, Novartis, Roche Pharma AG, Sanofi-Genzyme, TEVA, and WebMD Global. Prof. Wiendl is acting as a paid consultant for Abbvie, Actelion, Biogen, IGES, Novartis, Roche, Sanofi-Genzyme, and the Swiss Multiple Sclerosis Society. His research is funded by the German Ministry for Education and Research (BMBF; 01FI1601E, 01GI1603A, and O1GI1603D), Deutsche Forschungsgesellschaft (DFG; SFB128 A09, A10, Z02, V and SFB1009 A03), Else Kröner Fresenius Foundation, Fresenius Foundation, Hertie Foundation, NRW Ministry of Education and Research, Interdisciplinary Center for Clinical Studies (IZKF) Muenster and RE Children's Foundation, Biogen GmbH, GlaxoSmithKline GmbH, Roche Pharma AG, Sanofi-Genzyme.

The institution of BW receives honoraria for acting as a member of Scientific Advisory Boards for Biogen, Merck Serono, Roche, Sanofi-Genzyme, Novartis and speaker honoraria and travel support from Biogen, Merck Serono, Roche, Sanofi-Genzyme, Novartis, TEVA.

The other authors report no conflict of interest.

For peer review only - http://bmjopen.bmj.com/site/about/guidelines.xhtml

Study protocol for tolDC administration in patients with active MS

For peer review only

For peer review only - http://bmjopen.bmj.com/site/about/guidelines.xhtml

TABLES AND FIGURES

Table 1: In- and exclusion criteria. All the inclusion criteria must be fulfilled. The presence of any of the exclusion criteria shall exclude the patient from the study.

Inclusion criteria	Exclusion criteria
MS according to most recent McDonald criteria	Previous use of immunosuppressive or cytostatic treatment, including mitoxantrone, mavenclad, rituximab, alemtuzumab or bone marrow transplantation or stem cell transplantation at any time prior to enrolment
Age 18-60 years	Treatment with fingolimod or natalizumab or dimethylfumarate in the past 12 weeks or teriflunomide within the past 15 weeks or ocreluzimab within the past 6 months prior to the first administration
EDSS of 0-6.5 inclusive	Pregnancy or planning pregnancy in the next 12 months and breast feeding
First signs or symptoms at least 3 months prior to enrolment in the study	Drug or alcohol abuse

For peer review only

Active MS (relapsing and/or progressive): 1 relapse in the past year and/or at least 1 enhancing lesion on brain MRI in the past year; at least 1 new or enlarging T2 lesion in comparison with a reference scan from maximum 1 year before	Inability to undergo MRI assessments
Normal peripheral B-cell count after treatment with ocrelizumab	
No evidence of relapse for at least 30 days prior to start of screening and throughout during the screening phase	History of or actual signs of immunodeficiency or malignancies
Positive T cell reactivity response to a mix of 7 myelin-derived peptides	Concurrent clinically relevant cardiac, immunological, pulmonary, neurological, renal or other major disease
Able to sign informed consent and comply with the protocol assessments	Active or chronic infection (Hepatitis B or C, HIV, syphilis or tuberculosis)
No wish to be treated with currently available DMT	Splenectomy
Appropriate venous access and *adequate cervical lymph nodes on ultrasound	

For peer review only

mapping	
Use of adequate contraceptive measures. Women of childbearing potential can only be included in the study following use of adequate contraceptive measures. Accepted methods of contraception include use of hormonal contraceptives (oral, intravaginal, intrauterine, or transdermal), intrauterine devices, sterilization or postmenopausal status, use of condoms with spermicide.	

*only in TOLERVIT-MS

For peer review only

TABLE 2: Outline of the cell doses and patient numbers, per phase I clinical trial, in the dose escalation cohorts for intradermal and intranodal administration of tolDC. Abbreviations used: i.d., intradermal; tolDC, tolerogenic dendritic cells; N, number.

Cohort	Treatment Regimen	Patient numbers
**1**	6 i.d./i.n. injections of $5x10^6$ tolDC	N=3 (+1+1)
**2**	6 i.d./i.n. injections of $10x10^6$ tolDC	N=3 (+1+1)
**3**	6 i.d./i.n. injections of $15x10^6$ tolDC	N=3 (+1+1)

For peer review only

For peer review only - http://bmjopen.bmj.com/site/about/guidelines.xhtml

Study protocol for tolDC administration in patients with active MS

TABLE 3: Study calendar. The different visits, examinations, tests and the tolDC administration are detailed on the tabular study schedule overview.

	Screening		Treatment period							Safety follow up			
	Ts	Baseline (T0)	V1	V1 + 1day	V2	V3	V4	V5	V6	F1	F3	F6	F12
**Informed consent**	•												
**In- and exclusion criteria**	•												
**IFN-γ EliSPOT (T cell reactivity assay)**	•		•				•+ 1w		•+ 1w		•		
**Leukapheresis**		•											
**TolDC preparation**		•											
**TolDC treatment**			•		•	•	•	•	•				
**Patient evaluation:**													
- **Vital signs (HR, BP,…)**	•	•	•	•	•	•	•	•	•	•	•	•	•
- **ECG**	•	•	•	•	•	•	•	•	•	•	•	•	•
- **Blood analysis for safety (1x5ml EDTA tube and 2x10ml serum tubes)**	•	•	•	•	•	•	•	•	•	•	•	•	•
- **Urine pregnancy test**	•	•	•		•	•	•	•	•	•	•	•	•
**Full neurological examination (EDSS, 9-HPT, T25FW, SDMT, MSQOL-54)**	•		•				•		•	•	•	•	•
**Pain score after treatment injection**			•		•	•	•	•	•				
**MRI**	•	•	•		•		•+ 1w	•+ 1w	•+ 1w		•	•	•
**Immunomonitoring (10x10ml heparin tubes + 1 serum tube)**			•				•+ 1w		•+ 1w		•		
**Biobanking for follow up immunomonitoring**										•		•	•
**(Serious) Adverse events & concomitant medication**	Continuous												

For peer review only

Study protocol for tolDC administration in patients with active MS

Figure 1: Study design. Treatment will start at V_1, approximately 4 weeks after the leukapheresis. Patients will receive additional injections on week +2 (V_2), week +4 (V_3), week +6 (V_4), week +10 (V_5) and week +14 (V_6). Patients will have follow up visits one month, and 3, 6 and 12 months following the last study treatment.

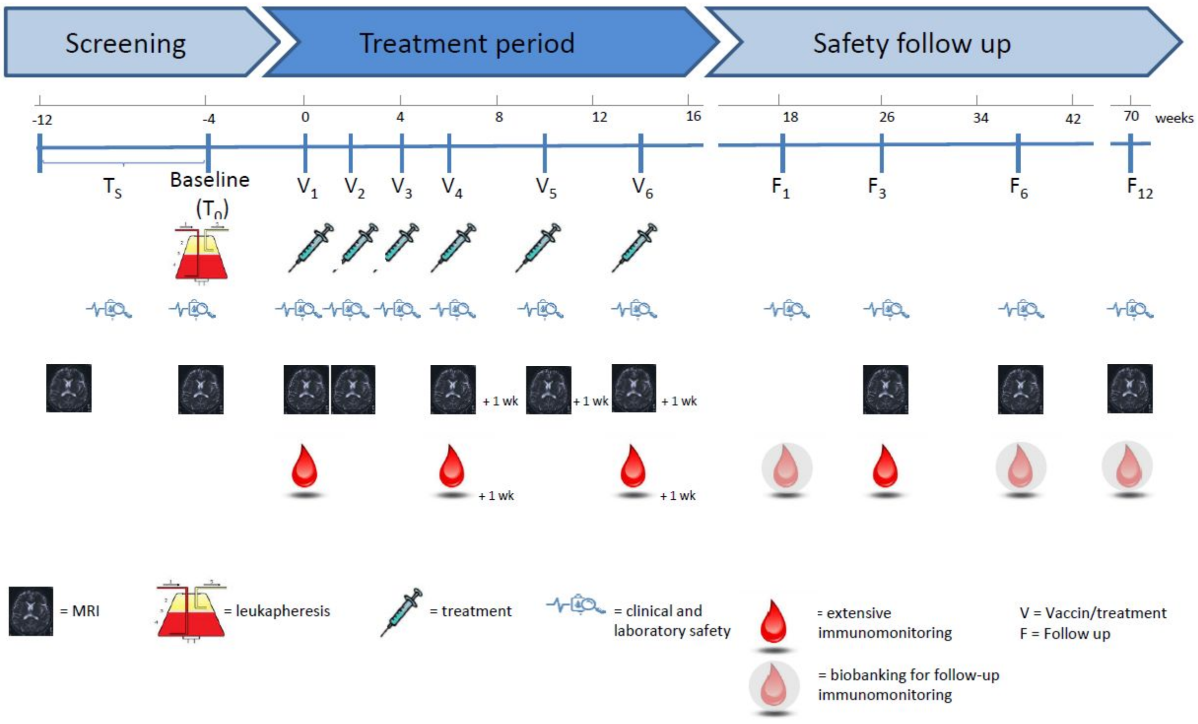

For peer review only - http://bmjopen.bmj.com/site/about/guidelines.xhtml

References

1. Lublin FD, Reingold SC, Cohen JA, et al. Defining the clinical course of multiple sclerosis: the 2013 revisions. *Neurology* 2014;83(3):278-86. doi: 10.1212/wnl.0000000000000560 [published Online First: 2014/05/30]
2. Scalfari A, Neuhaus A, Daumer M, et al. Onset of secondary progressive phase and long-term evolution of multiple sclerosis. *Journal of neurology, neurosurgery, and psychiatry* 2014;85(1):67-75. doi: 10.1136/jnnp-2012-304333 [published Online First: 2013/03/15]
3. Cree BA, Gourraud PA, Oksenberg JR, et al. Long-term evolution of multiple sclerosis disability in the treatment era. *Annals of neurology* 2016;80(4):499-510. doi: 10.1002/ana.24747 [published Online First: 2016/07/28]
4. Scolding N, Barnes D, Cader S, et al. Association of British Neurologists: revised (2015) guidelines for prescribing disease-modifying treatments in multiple sclerosis. *Practical neurology* 2015;15(4):273-9. doi: 10.1136/practneurol-2015-001139 [published Online First: 2015/06/24]
5. Dendrou CA, Fugger L, Friese MA. Immunopathology of multiple sclerosis. *Nature reviews Immunology* 2015;15(9):545-58. doi: 10.1038/nri3871 [published Online First: 2015/08/08]
6. Grigoriadis N, van Pesch V. A basic overview of multiple sclerosis immunopathology. *Eur J Neurol* 2015;22 Suppl 2:3-13. doi: 10.1111/ene.12798 [published Online First: 2015/09/17]
7. Bielekova B, Goodwin B, Richert N, et al. Encephalitogenic potential of the myelin basic protein peptide (amino acids 83-99) in multiple sclerosis: results of a phase II clinical trial with an altered peptide ligand. *Nature medicine* 2000;6(10):1167-75. doi: 10.1038/80516 [published Online First: 2000/10/04]
8. Bielekova B, Sung MH, Kadom N, et al. Expansion and functional relevance of high-avidity myelin-specific CD4+ T cells in multiple sclerosis. *Journal of immunology (Baltimore, Md : 1950)* 2004;172(6):3893-904. [published Online First: 2004/03/09]
9. Wallstrom E, Khademi M, Andersson M, et al. Increased reactivity to myelin oligodendrocyte glycoprotein peptides and epitope mapping in HLA DR2(15)+ multiple sclerosis. *European journal of immunology* 1998;28(10):3329-35. doi: 10.1002/(sici)1521-4141(199810)28:10<3329::aid-immu3329>3.0.co;2-b [published Online First: 1998/11/10]
10. Grau-Lopez L, Raich D, Ramo-Tello C, et al. Specific T-cell proliferation to myelin peptides in relapsing-remitting multiple sclerosis. *Eur J Neurol* 2011;18(8):1101-4. doi: 10.1111/j.1468-1331.2010.03307.x [published Online First: 2011/07/14]
11. Van Brussel I, Lee WP, Rombouts M, et al. Tolerogenic dendritic cell vaccines to treat autoimmune diseases: can the unattainable dream turn into reality? *Autoimmunity reviews* 2014;13(2):138-50. doi: 10.1016/j.autrev.2013.09.008 [published Online First: 2013/10/15]
12. Willekens B, Cools N. Beyond the Magic Bullet: Current Progress of Therapeutic Vaccination in Multiple Sclerosis. *CNS drugs* 2018 doi: 10.1007/s40263-018-0518-4 [published Online First: 2018/05/16]
13. Gonsette RE. Self-tolerance in multiple sclerosis. *Acta Neurol Belg* 2012;112(2):133-40. doi: 10.1007/s13760-012-0061-x [published Online First: 2012/03/28]
14. Lutterotti A, Martin R. Antigen-specific tolerization approaches in multiple sclerosis. *Expert opinion on investigational drugs* 2014;23(1):9-20. doi: 10.1517/13543784.2014.844788 [published Online First: 2013/10/25]
15. Gross CC, Jonuleit H, Wiendl H. Fulfilling the dream: tolerogenic dendritic cells to treat multiple sclerosis. *European journal of immunology* 2012;42(3):569-72. doi: 10.1002/eji.201242402 [published Online First: 2012/04/11]
16. Berneman Z, Lee W, Willekens B, et al. Towards the Clinical Use of Tolerogenic Dendritic Cells in Multiple Sclerosis by Applying the Immunomodulatory Effects of 1, 25-dihydroxyvitamin D3 on Dendritic Cells to Promote Induction of T Cell Hyporesponsiveness to Myelin-Derived Antigens. *Cytotherapy* 2016;18(6):S95-S96.

For peer review only

17. Using cells to cure disease: Tolerogenic dendritic cells for the treatment of MS. MULTIPLE SCLEROSIS JOURNAL; 2016. SAGE PUBLICATIONS LTD 1 OLIVERS YARD, 55 CITY ROAD, LONDON EC1Y 1SP, ENGLAND.
18. Ten Brinke A, Hilkens CM, Cools N, et al. Clinical Use of Tolerogenic Dendritic Cells-Harmonization Approach in European Collaborative Effort. *Mediators of inflammation* 2015;2015:471719. doi: 10.1155/2015/471719 [published Online First: 2016/01/29]
19. Nuyts AH, Lee WP, Bashir-Dar R, et al. Dendritic cells in multiple sclerosis: key players in the immunopathogenesis, key players for new cellular immunotherapies? *Multiple sclerosis (Houndmills, Basingstoke, England)* 2013;19(8):995-1002. doi: 10.1177/1352458512473189 [published Online First: 2013/02/02]
20. Florez-Grau G, Zubizarreta I, Cabezon R, et al. Tolerogenic Dendritic Cells as a Promising Antigen-Specific Therapy in the Treatment of Multiple Sclerosis and Neuromyelitis Optica From Preclinical to Clinical Trials. *Frontiers in immunology* 2018;9:1169. doi: 10.3389/fimmu.2018.01169 [published Online First: 2018/06/16]
21. Lee W-P, Willekens B, Cras P, et al. Immunomodulatory Effects of 1, 25-Dihydroxyvitamin D3 on Dendritic Cells Promote Induction of T Cell Hyporesponsiveness to Myelin-Derived Antigens. *Journal of Immunology Research* 2016;2016
22. Raich-Regue D, Grau-Lopez L, Naranjo-Gomez M, et al. Stable antigen-specific T-cell hyporesponsiveness induced by tolerogenic dendritic cells from multiple sclerosis patients. *European journal of immunology* 2012;42(3):771-82. doi: 10.1002/eji.201141835 [published Online First: 2012/04/11]
23. Naranjo-Gomez M, Raich-Regue D, Onate C, et al. Comparative study of clinical grade human tolerogenic dendritic cells. *Journal of translational medicine* 2011;9:89. doi: 10.1186/1479-5876-9-89 [published Online First: 2011/06/11]
24. Mansilla MJ, Selles-Moreno C, Fabregas-Puig S, et al. Beneficial effect of tolerogenic dendritic cells pulsed with MOG autoantigen in experimental autoimmune encephalomyelitis. *CNS neuroscience & therapeutics* 2015;21(3):222-30. doi: 10.1111/cns.12342 [published Online First: 2014/11/19]
25. Mansilla MJ, Contreras-Cardone R, Navarro-Barriuso J, et al. Cryopreserved vitamin D3-tolerogenic dendritic cells pulsed with autoantigens as a potential therapy for multiple sclerosis patients. *Journal of neuroinflammation* 2016;13(1):113. doi: 10.1186/s12974-016-0584-9 [published Online First: 2016/05/22]
26. Giannoukakis N, Phillips B, Finegold D, et al. Phase I (safety) study of autologous tolerogenic dendritic cells in type 1 diabetic patients. *Diabetes care* 2011;34(9):2026-32. doi: 10.2337/dc11-0472 [published Online First: 2011/06/18]
27. Benham H, Nel HJ, Law SC, et al. Citrullinated peptide dendritic cell immunotherapy in HLA risk genotype-positive rheumatoid arthritis patients. *Science translational medicine* 2015;7(290):290ra87. doi: 10.1126/scitranslmed.aaa9301 [published Online First: 2015/06/05]
28. Bell GM, Anderson AE, Diboll J, et al. Autologous tolerogenic dendritic cells for rheumatoid and inflammatory arthritis. *Annals of the rheumatic diseases* 2017;76(1):227-34. doi: 10.1136/annrheumdis-2015-208456 [published Online First: 2016/04/28]
29. Jauregui-Amezaga A, Cabezon R, Ramirez-Morros A, et al. Intraperitoneal Administration of Autologous Tolerogenic Dendritic Cells for Refractory Crohn's Disease: A Phase I Study. *Journal of Crohn's & colitis* 2015;9(12):1071-8. doi: 10.1093/ecco-jcc/jjv144 [published Online First: 2015/08/26]
30. De Vries IJ, Krooshoop DJ, Scharenborg NM, et al. Effective migration of antigen-pulsed dendritic cells to lymph nodes in melanoma patients is determined by their maturation state. *Cancer research* 2003;63(1):12-7. [published Online First: 2003/01/09]
31. Ridolfi R, Riccobon A, Galassi R, et al. Evaluation of in vivo labelled dendritic cell migration in cancer patients. *Journal of translational medicine* 2004;2(1):27. doi: 10.1186/1479-5876-2-27 [published Online First: 2004/08/03]

For peer review only - http://bmjopen.bmj.com/site/about/guidelines.xhtml

32. Morse MA, Coleman RE, Akabani G, et al. Migration of human dendritic cells after injection in patients with metastatic malignancies. *Cancer research* 1999;59(1):56-8. [published Online First: 1999/01/19]
33. Lesterhuis WJ, de Vries IJ, Schreibelt G, et al. Route of administration modulates the induction of dendritic cell vaccine-induced antigen-specific T cells in advanced melanoma patients. *Clinical cancer research : an official journal of the American Association for Cancer Research* 2011;17(17):5725-35. doi: 10.1158/1078-0432.ccr-11-1261 [published Online First: 2011/07/21]
34. de Vries IJ, Lesterhuis WJ, Barentsz JO, et al. Magnetic resonance tracking of dendritic cells in melanoma patients for monitoring of cellular therapy. *Nature biotechnology* 2005;23(11):1407-13. doi: 10.1038/nbt1154 [published Online First: 2005/11/01]
35. Polman CH, Reingold SC, Banwell B, et al. Diagnostic criteria for multiple sclerosis: 2010 revisions to the McDonald criteria. *Annals of neurology* 2011;69(2):292-302. doi: 10.1002/ana.22366 [published Online First: 2011/03/10]
36. Thompson AJ, Banwell BL, Barkhof F, et al. Diagnosis of multiple sclerosis: 2017 revisions of the McDonald criteria. *The Lancet Neurology* 2018;17(2):162-73. doi: 10.1016/s1474-4422(17)30470-2 [published Online First: 2017/12/26]
37. Le Tourneau C, Lee JJ, Siu LL. Dose escalation methods in phase I cancer clinical trials. *Journal of the National Cancer Institute* 2009;101(10):708-20. doi: 10.1093/jnci/djp079 [published Online First: 2009/05/14]
38. Kurtzke JF. Rating neurologic impairment in multiple sclerosis: an expanded disability status scale (EDSS). *Neurology* 1983;33(11):1444-52. [published Online First: 1983/11/01]
39. Vickrey BG, Hays RD, Harooni R, et al. A health-related quality of life measure for multiple sclerosis. *Quality of life research : an international journal of quality of life aspects of treatment, care and rehabilitation* 1995;4(3):187-206. [published Online First: 1995/06/01]
40. Nafarrate IZ, Florez G, Vila G, et al. Phase 1b clinical trial with antigen-specific tolerogenic dendritic in Multiple Sclerosis and Neuromyelitis optica: safety and immunological effects (P2.330). *Neurology* 2017;88(16 Supplement):P2.330.

For peer review only

For peer review only - http://bmjopen.bmj.com/site/about/guidelines.xhtml

BMJ Open

Tolerogenic dendritic cell-based treatment for multiple sclerosis (MS): a harmonized study protocol for two phase I clinical trials comparing intradermal and intranodal cell administration

Journal:	*BMJ Open*
Manuscript ID	bmjopen-2019-030309.R1
Article Type:	Protocol
Date Submitted by the Author:	09-Jul-2019
Complete List of Authors:	Willekens, Barbara; University Hospital Antwerp, Department of Neurology ; University of Antwerp Faculty of Medicine and Health Sciences, Laboratory of Experimental Hematology, Vaccine & Infectious Disease Institute (VAXINFECTIO)<br>Presas-Rodríguez, Silvia ; Hospital Universitari Germans Trias i Pujol, Multiple Sclerosis Unit, Department of Neurosciences; Universitat Autònoma de Barcelona, Department of Medicine<br>Mansilla, MJ; Hospital Universitario Germans Trias i Pujol and Research Institute, Division of Immunology, LCMN; Universitat Autònoma de Barcelona, Department of Cellular Biology, Physiology and Immunology<br>Derdelinckx, Judith; University Hospital Antwerp, Neurology ; University of Antwerp Faculty of Medicine and Health Sciences, Laboratory of Experimental Hematology , Vaccine & Infectious Disease Institute (VAXINFECTIO)<br>Lee, Wai-Ping; University Hospital Antwerp, Center for Cell Therapy and Regenerative Medicine<br>Nijs, Griet; University Hospital Antwerp, Center for Cell Therapy and Regenerative Medicine<br>De Laere, Maxime; University of Antwerp Faculty of Medicine and Health Sciences, Laboratory of Experimental Hematology, Vaccine & Infectious Disease Institute (VAXINFECTIO)<br>Wens, Inez; University of Antwerp Faculty of Medicine and Health Sciences, Laboratory of Experimental Hematology, Vaccine & Infectious Disease Institute (VAXINFECTIO)<br>Cras, Patrick ; University Hospital Antwerp, Department of Neurology<br>Parizel, Paul; University Hospital Antwerp, Department of Radiology; Icometrix NV<br>Van Hecke, Wim; Icometrix NV<br>Ribbens, Annemie; Icometrix NV<br>Billiet, Thibo; Icometrix NV<br>Adams, Geert; C-Clear Partners<br>Couttenye, marie-madeleine; University Hospital Antwerp, Department of Nephrology<br>Navarro-Barriuso, Juan; Hospital Universitario Germans Trias i Pujol and Research Institute, Division of Immunology, LCMN; Universitat Autònoma de Barcelona, Department of Cellular Biology, Physiology and Immunology<br>Teniente-Serra , Aina; Hospital Universitario Germans Trias i Pujol and

Research Institute, Division of Immunology, LCMN; Universitat Autònoma de Barcelona, Department of Cellular Biology, Physiology and Immunology
Quirant-Sánchez , Bibiana; Hospital Universitario Germans Trias i Pujol and Research Institute, Division of Immunology, LCMN; Universitat Autònoma de Barcelona, Department of Cellular Biology, Physiology and Immunology
Lopez-Diaz de Cerio, A; clinica universidad de navarra, Haematology-Cell Therapy Area; clinica universidad de navarra, Immunology and Immunotherapy Department
Inogés, S; clinica universidad de navarra, Haematology-Cell Therapy Area; clinica universidad de navarra, Immunology and Immunotherapy Department
Prosper, Felipe; clinica universidad de navarra, Haematology-Cell Therapy Area; Universidad de Navarra, Program of Haematology-Oncology, CIMA
Kip, Anke; Lygature
Verheij, Herman; Lygature
Gross, CC; University Hospital Munster, Department of Neurology; University of Munster, Department of Neurology
Wiendl, Heinz; University Hospital Munster, Department of Neurology; University of Munster, Department of Neurology
Van Ham, SM; Sanquin Research, Department of Immunopathology; University of Amsterdam, Landsteiner Laboratory, Academic Medical Centre
Ten Brinke, Anja; Sanquin Research, Department of Immunopathology; University of Amsterdam, Landsteiner Laboratory, Academic Medical Centre
Barriocanal, Ana Maria; Germans Trias i Pujol Health Sciences Research Institute , Clinical Research Polyvalent Unit, Clinial Trial Unit-Spanish Clinical Research Network ; Universitat Autònoma de Barcelona, Department of Pharmacology, Therapeutic and Toxicology
Massuet-Vilamajó , A; Hospital Universitari Germans Trias i Pujol, Institut de Diagnòstic per la Imatge
Hens, Niel; Universiteit Hasselt, Interuniversity Institute for Biostatistics and statistical Bioinformatics (I-BioStat); University of Antwerp Faculty of Medicine and Health Sciences, Centre for Health Economic Research and Modelling Infectious Diseases, Vaccine & Infectious Disease Institute (VAXINFECTIO)& Center for Statistics
Berneman, Zwi; University of Antwerp Faculty of Medicine and Health Sciences, Laboratory of Experimental Hematology ; University Hospital Antwerp, Center for Cell Therapy and Regenerative Medicine
Martínez-Cáceres , Eva; Hospital Universitario Germans Trias i Pujol and Research Institute, Division of Immunology, LCMN
Cools, Nathalie; University of Antwerp Faculty of Medicine and Health Sciences, Laboratory of Experimental Hematology, Vaccine & Infectious Disease Institute (VAXINFECTIO); University Hospital Antwerp, Center for Cell Therapy and Regenerative Medicine
Ramo-Tello, Cristina; Hospital Universitari Germans Trias i Pujol, Multiple Sclerosis Unit, Department of Neurosciences

**Primary Subject Heading**:	Neurology
Secondary Subject Heading:	Immunology (including allergy), Patient-centred medicine, Radiology and imaging, Research methods
Keywords:	Multiple sclerosis < NEUROLOGY, Clinical trials < THERAPEUTICS, Immunology < BASIC SCIENCES, IMMUNOLOGY, Magnetic resonance imaging < RADIOLOGY & IMAGING

Tolerogenic dendritic cell-based treatment for multiple sclerosis (MS): a harmonized study protocol for two phase I clinical trials comparing intradermal and intranodal cell administration

Willekens B[$,1,2], Presas-Rodríguez, S[$,3,4], Mansilla MJ[5,6], Derdelinckx J[1,2], Lee W-P[7], Nijs G[7], De Laere M[2], Wens I[2], Cras P[1], Parizel PM[8,9], Van Hecke W[9], Ribbens A[9], Billiet T[9], Adams G[10], Couttenye M[11], Navarro-Barriuso J[5,6], Teniente-Serra A[5,6], Quirant-Sánchez B[5,6], López-Díaz de Cerio A[12,13], Inogés S[12,13], Prósper F[12,14], Kip A[15], Verheij H[15], Gross CC[16], Wiendl H[16], van Ham SM[17], ten Brinke A[17], Barriocanal AM[18,19], Massuet-Vilamajó A[20], Hens N[21], Berneman Z[2,7], Martínez-Cáceres EM[5], Cools N[#,2,7*] and Ramo-Tello C[#,3] on behalf of the RESTORE consortium

[$]both first authors contributed equally to this paper

[#]both senior authors contributed equally to this paper

*corresponding author: Nathalie.Cools@uza.be and cramot@gmail.com

Author affiliations:

1 Department of Neurology, Antwerp University Hospital, Edegem, Belgium

2 Laboratory of Experimental Hematology, Vaccine & Infectious Disease Institute (VAXINFECTIO), Faculty of Medicine and Health Sciences, University of Antwerp, Wilrijk, Belgium

3 Multiple Sclerosis Unit, Department of Neurosciences, Hospital Universitari Germans Trias i Pujol, Badalona, Spain

4 Department of Medicine, Universitat Autònoma de Barcelona, Cerdanyola del Vallés, Spain

For peer review only

5 Division of Immunology,LCMN, Hospital Universitario Germans Trias i Pujol and Research Institute, Campus Can Ruti, Badalona, Spain

6 Department of Cellular Biology, Physiology and Immunology, Universitat Autònoma de Barcelona, Bellaterra, Spain

7 Center for Cell Therapy and Regenerative Medicine, Antwerp University Hospital, Edegem, Belgium

8 Department of Radiology, Antwerp University Hospital, Edegem, Belgium

9 Icometrix, Kolonel Begaultlaan 1b, Leuven, Belgium

10 C-Clear Partners, Mortsel, Belgium

11 Department of Nephrology, Antwerp University Hospital, Edegem, Belgium

12 Haematology-Cell Therapy Area, Clinica Universidad de Navarra, Pamplona, Spain

13 Immunology and Immunotherapy Department, Clinica Universidad de Navarra, Pamplona, Spain

14 Program of Haematology-Oncology, CIMA, Universidad de Navarra, Pamplona, Spain

15 Lygature, Utrecht, The Netherlands

16 Department of Neurology, University Hospital and University Münster, Münster, Germany

17 Department of Immunopathology, Sanquin Research, Amsterdam, The Netherlands and Landsteiner Laboratory, Academic Medical Centre, University of Amsterdam, Amsterdam, The Netherlands

18 Clinical Research Polyvalent Unit, Germans Trias i Pujol Health Sciences Research Institute (IGTP), Badalona, Spain. Clinial Trial Unit-Spanish Clinical Research Network SCReN.

19 Department of Pharmacology, Therapeutic and Toxicology, Universitat Autònoma de Barcelona, Cerdanyola del Vallès, Spain

20 Institut de Diagnòstic per la Imatge, Hospital Universitari Germans Trias i Pujol, Badalona, Spain

21 Centre for Health Economic Research and Modelling Infectious Diseases, Vaccine & Infectious Disease Institute (VAXINFECTIO), Wilrijk, Belgium & Center for Statistics, I-BioStat, Hasselt University, Hasselt, Belgium

For peer review only

For peer review only - http://bmjopen.bmj.com/site/about/guidelines.xhtml

ABSTRACT

Introduction: Based on the advances in the treatment of multiple sclerosis (MS), currently available disease-modifying treatments (DMT) have positively influenced the disease course of MS. However, the efficacy of DMT is highly variable and increasing treatment efficacy comes with a more severe risk profile. Hence, the unmet need for safer and more selective treatments remains. Specifically restoring immune tolerance towards myelin antigens may provide an attractive alternative. In this respect, antigen-specific tolerization with autologous tolerogenic dendritic cells (tolDC) is a promising approach.

Methods and analysis: Here, we will evaluate the clinical use of tolDC in a well-defined population of MS patients in two phase I clinical trials. In doing so, we aim to compare two ways of tolDC administration, namely intradermal and intranodal. The cells will be injected at consecutive intervals in 3 cohorts receiving incremental doses of tolDC, according to a best-of-five design. The primary objective is to assess the safety and feasibility of tolDC administration. For safety, the number of adverse events (AE) including magnetic resonance imaging (MRI) and clinical outcomes will be assessed. For feasibility, successful production of tolDC will be determined. Secondary endpoints include clinical and MRI outcome measures. The patients' immune profile will be assessed to find presumptive evidence for a tolerogenic effect *in vivo*.

Ethics and dissemination: Ethics approval was obtained for the two phase I clinical trials. The results of the trials will be disseminated in a peer-reviewed journal, at scientific conferences and to patient associations.

Trial registration numbers: Both studies are registered at clinicaltrials.gov: NCT02618902 and NCT02903537; EudraCT numbers are 2015-002975-16 and 2015-003541-26, respectively.

For peer review only

For peer review only - http://bmjopen.bmj.com/site/about/guidelines.xhtml

STRENGTHS AND LIMITATIONS OF THIS STUDY

- The concept of the clinical trials is built on recent advances in the understanding of tolerance induction via cell-based therapy, and the effort made to precisely silence myelin-antigen-specific, putatively deleterious immune responses in the disease.
- The use of cryopreserved tolDC allows production of batches of tolDC, stored in ready-to-use aliquots for the required administrations during the treatment period, thereby reducing variability and global production cost.
- Harmonization of clinical, MRI and immunological evaluations of the patients will enable us to compare results between two phase I clinical trials evaluating the safety and feasibility of autologous tolDC administration in patients with active MS.
- The patients are not randomized across the trials, limiting direct comparison of both routes of administration.

For peer review only

INTRODUCTION

Multiple sclerosis (MS) is the leading cause of non-traumatic disability in young adults and typically presents between the age of 20-40 years, in the prime of a patient's personal and professional life. The heterogeneity of the disease course in MS, i.e. relapsing-remitting or progressive, remains a challenge for patient management and design of clinical trials. According to Lublin et al.[1], MS is categorized as relapsing or progressive. In both forms, disease activity (defined by clinical relapse and/or MRI lesions) and disease progression are measured. To date, more than 10 disease-modifying treatments (DMT) are approved for clinical use in patients with active relapsing-remitting MS (RRMS). This has dramatically influenced the management of MS patients in daily practice and has positively influenced prognosis of of a subset of MS patients. Indeed, whereas earlier epidemiological studies indicated that 50% of patients develop secondary progressive MS (SPMS) after 15 years when untreated[2], only 18% of patients treated with modern MS therapeutics were reported to evolve to a secondary progressive course after a median duration of 16.8 years in a recent cohort[3]. Nonetheless, DMT are not specific nor selective for MS, i.e. they work in an immunomodulatory or immunosuppressive way by sequestering or depleting lymphocytes, albeit that some therapies can induce immune reconstitution as well. Moreover, treatment-related side effects or risks can be severe[4], leaving a significant and unmet need for safer and more disease-selective treatments.

The crux of MS is the patient's own immune cells attacking self-antigens in the central nervous system (CNS), caused by a loss of tolerance against myelin antigens. This is manifested by inflammatory infiltrates, demyelination and axonal loss, resulting in the clinical symptoms of the disease[5 6]. Previously, it was shown that proteins expressed in the myelin sheath, protecting neuronal axons of the CNS, are an important target of the

For peer review only

For peer review only - http://bmjopen.bmj.com/site/about/guidelines.xhtml

autoreactive T cell response[7-9]. Also in our hands, *ex vivo* T cell reactivity against a mix of 7 immunodominant myelin-derived peptides could be demonstrated in RRMS patients as compared to healthy controls and patients with other neurological disorders[10]. Although the underlying cause of the loss of tolerance towards myelin antigens has not been elucidated yet, one ultimate aim in the treatment of MS is to re-establish antigen-specific immune tolerance towards CNS structures[11-15]. In this perspective, it is postulated that tolerance-inducing antigen-specific therapy can be an innovative and promising strategy for the treatment of autoimmunity. This approach is based on the possibility that autoreactive B and T cells, driving myelin destruction and damage in the CNS, will be eradicated by inducing tolerance to myelin-derived peptides, without interfering with protective immunity. Several approaches involving the induction of antigen-specific tolerance have reached the clinical development phase and demonstrated promising results in phase I/II clinical trials (extensively reviewed by Willekens and Cools[12]). Given the fact that dendritic cells (DC) play a key role in controlling the immune response by steering the outcome of antigen presentation to T cells, the use of tolerance-inducing or tolerogenic dendritic cells (tolDC) may provide prospect for the treatment of MS[16-20]. Recently, we demonstrated the potential of 1α,25-dihydroxyvitamin D_3 to generate tolDC from MS patients[21-23]. Indeed, vitamin D_3-treated tolDC from MS patients display a maturation-resistant phenotype, i.e. they maintain their low expression levels of costimulatory molecules (CD80, CD83 and CD86) and anti-inflammatory cytokine profile, even following re-challenge with an inflammatory stimulus such as lipopolysaccharide (LPS). Furthermore, tolDC from MS patients are capable of inducing stable and antigen-specific T cell hyporesponsiveness. After *in vitro* stimulation with myelin-derived peptide-pulsed vitamin D_3-treated tolDC, T cells were unresponsive to the myelin peptides used, while retaining their capacity to respond to an unrelated antigen.

For peer review only - http://bmjopen.bmj.com/site/about/guidelines.xhtml

134 Moreover, T cell hyporesponsiveness was robust, as T cells were not reactivated after
135 rechallenge with immunostimulatory DC[21-23].
136 Also *in vivo*, the administration of bone marrow-derived vitamin D_3-treated tolDC pulsed
137 with MOG_{40-55} has been shown to induce antigen-specific T cell tolerance in experimental
138 autoimmune encephalomyelitis (EAE), the animal model of MS. Mice treated with MOG_{40-55}-
139 pulsed bone marrow-derived vitamin D_3-treated tolDC before disease induction showed
140 reduced incidence of the disease. Furthermore, when the treatment was used
141 therapeutically in mice already showing clinical signs of the disease, the severity of the
142 disease was significantly reduced[24]. However, repetitive injections of tolDC were necessary
143 for a prolonged clinical effect in EAE. In this context, cryopreserved MOG_{40-55}-pulsed tolDC
144 demonstrated similar clinical benefit compared to fresh tolDC, underlining the therapeutic
145 potential and clinical applicability of cryopreserved tolDC[21 25].
146 Recently, a number of phase I studies investigating the safety and feasibility of tolDC therapy
147 for autoimmune diseases such as rheumatoid arthritis, type I diabetes, or Crohn's disease
148 were completed (Table 1)[18]. The first results are highly encouraging since none of the trials
149 found safety concerns related to tolDC administration in these patients. TolDC were well
150 tolerated and autoimmunity was not exacerbated in the patients treated[26-29]. Nevertheless,
151 numerous questions remain, and the efficacy of antigen-specific tolDC therapy may depend
152 on many factors, of which the route of administration is among the most important. When
153 considering the route of delivery of DC, one needs to take into account that different routes
154 lead to different sites of accumulation of the vaccinated DC. In most clinical studies to date,
155 *ex vivo* generated DC were injected either intravenously, intradermally or subcutaneously. In
156 humans, migration of DC towards the secondary lymph nodes is superior after intradermal
157 injection compared with after subcutaneous injection of DC[30-32], whereas migration of

For peer review only - http://bmjopen.bmj.com/site/about/guidelines.xhtml

intravenously injected DC has not been monitored so far. Direct delivery of DC to lymph nodes via intranodal injection showed promising results in therapies using immunostimulatory DC in cancer[33 34], but has not been evaluated for tolDC. It was demonstrated that brain-derived antigens can be drained from the interstitial or cerebrospinal fluid via the *lamina cribrosa* and nasal mucosa to the cervical lymph nodes, indicating that the cervical lymph nodes could be one of the first stations for the antigenic presentation at the peripheral level.[35 36] Hence, we hypothesize that intranodal injection of tolDC directly interferes with the antigen presentation and consequently, the stimulation and proliferation of autoreactive T cells. Furthermore, this route of administration omits the need for the migration requirements of the tolDC, thereby potentially enhancing the efficacy of the vaccine. Although intranodal injection is more complex, requiring ultrasound guidance, this technique is part of the daily practice at the endocrinology or radiology department of most reference hospitals.

Until now, there are no available data showing superiority of one route over others for the administration of peptide-loaded tolDC. Here, we will compare intradermal (i.d.) injection to intranodal (i.n.) injection. Only the direct comparison of these different routes of administration in two dose-escalation studies will allow us to determine if both routes are equally safe.

In conclusion, our objectives are to evaluate safety, clinical feasibility and the immunological consequences of peptide-loaded tolDC administered intranodal or intradermal in MS patients in two clinical trials. Harmonization of the procedures for clinical, MRI as well as immune-monitoring will enable us to compare results between trials of which the study protocols will be discussed in detail here.

For peer review only - http://bmjopen.bmj.com/site/about/guidelines.xhtml

METHODS AND ANALYSIS

Study design

Two open-label, dose-escalation phase I clinical trials, MS-tolDC and TOLERVIT-MS, are designed in a coordinated and comprehensive manner and run simultaneously in Belgium (Antwerp University Hospital, Edegem) and Spain (Hospital Germans Trias i Pujol, Badalona), respectively. Ethics approval was obtained for both phase I clinical trials. Patient recruitment started mid-2017 and is anticipated to end in 2019.

Primary objectives

To evaluate the safety of administering tolDC, the occurrence and severity of adverse events (AE) will be recorded. To assess feasibility, successful production of tolDC according to good manufacturing practices (GMP) starting from a leukapheresis procedure will be assessed.

Secondary objectives

Preliminary efficacy measures, including clinical outcomes and brain MRI, will be evaluated. In addition, whole blood lymphocyte phenotyping and cytokine profiling will be assessed before and after completion of the vaccination cycle, as well as the ability of tolDC to suppress pathogenic T cell responses. For this, myelin-specific T cell reactivity will be determined before and after completion of the vaccination cycle. All secondary objectives contribute to determination of proof of principle.

Patient-reported outcomes

MS QOL-54 will be evaluated to detect changes in general and disease-specific quality of life.

Patient and public involvement

For peer review only

This study was inspired by the unmet need of finding a cure for MS, which is a key priority recognized by both patients and neurologists[37]. While patients were not involved in the design of the clinical trial, they are involved in the conduct of the study. In fact, in order to spread awareness and information about the clinical trials and its results beyond the research community with the public as a whole, a stakeholder committee was installed consisting of experts from major interest groups including members of the Flemish, Belgian and Spanish MS societies as well as Flemish and Spanish MS patients. This stakeholder committee will accompany the clinical trials from the start to the end. It has several aims: (i) to keep the stakeholder informed, (ii) to ensure that their views are considered, (iii) to challenge the project by the identification of potential emerging needs, and (iv) to play an active part in the dissemination and the use of the project's results.

Study population

Eligibility and enrolment

Patients with active relapsing-remitting and active progressive MS, diagnosed according to most recent McDonald criteria[38 39], and who are not eligible for or do not want to be treated with currently available DMT, will be recruited. Patients are included after written informed consent and enrolled in the study when the in- and exclusion criteria are met (Table 2).

Determination of sample size and dose-escalation procedure

The studies will be conducted according to a "best of five" design[40], an alternative of the traditional 3+3 design in that one additional patient is added when one or even two dose-limiting toxicities (DLT) are observed among the first three patients. Another patient is added when two DLT are observed among four treated patients. Dose escalation is allowed if DLT are observed among none of three, one of four, or two of five patients, but the trial will terminate if three or more DLT are observed. A DLT is defined as a serious adverse event

For peer review only

(SAE) that is attributable to the study cells administered, or of which the severity prevents further escalation. Dose escalation decisions will be made after all subjects in the cohort have completed at least 3 months of follow up and when the results of the safety and tolerability analyses of the preceding dose regimen are satisfactory in the judgment of the investigators and the independent Data Safety Monitoring Board (DSMB).

An overview of the dose escalation is provided in Table 3. Altogether, each phase I study is intended to accrue a total number of 9-15 evaluable patients.

Study medication

Generation of tolDC will be carried out according to the principles and guidelines of GMP laid down in Directive 2003/94/EC. TolDC production will be performed in the GMP facilities of the Center for Cell Therapy and Regenerative Medicine (CCRG) of the Antwerp University Hospital (Belgium) and of the Cell Therapy Area of the Clínica Universidad de Navarra (Spain).

Clinical-grade autologous tolDC will be prepared from a leukapheresis. $CD14^+$ monocytes will be cultured in GMP-grade cell culture medium supplemented with 2% human AB serum, granulocyte macrophage colony-stimulating factor (GM-CSF), interleukin (IL)-4 and 1α,25 dihydroxyvitamin D_3. At day 4, tolDC will be stimulated using an inflammatory cytokine cocktail, consisting of tumor necrosis factor (TNF)-α, prostaglandin (PG) E_2 and IL-1β. At day 6, tolDC will be harvested, loaded with 7 myelin antigens (MBP_{13-32}, $MBP_{111-129}$, $MBP_{154-170}$, $PLP_{139-154}$, MOG_{1-20}, MOG_{35-55} and MBP_{83-99}), and cryopreserved at -196°C. Separate aliquots of the cell product will be prepared for quality control and quality assurance (QC/QA). This includes (i) sterility testing, (ii) cell count, (iii) viability, (iv) flow cytometric phenotyping, and

For peer review only

(v) induction of T cell hyporesponsiveness in an allogeneic mixed leukocyte reaction (allo-MLR).

Trial intervention: tolDC administration

In this study, 6 vaccine doses will be administered to each participant according to the following immunization schedule: a total of 6 vaccine doses (V), with V_{1-4} at biweekly (± 3 days) intervals and V_{5-6} at monthly intervals (± 3 days). A complete overview of the study design is depicted in Figure 1. Detailed assessments per visit are shown in Table 4.

In Belgium (MS-tolDC), tolDC vaccination occurs through intradermal injection at 5 alternating sites (100µL/site) in the posterior neck region to ensure lymphatic drainage to superficial and deep cervical lymph nodes (5-10 cm from the cervical lymph nodes). In addition, in Spain (TOLERVIT-MS), an expert physician will inject tolDC directly in the cervical lymph nodes under echographic guidance.. If the patient's anatomical conditions do not allow it (e.g., small lymph nodes), the vaccine will be distributed in more nodes (max. 500µL/node).

For peer review only - http://bmjopen.bmj.com/site/about/guidelines.xhtml

Primary outcome measures

Safety

To evaluate the safety of administered tolDC, occurrence of AE will be recorded using clinical outcome measures, i.e. physical (skin, pain, adenopathies) and neurological examination (relapses, worsening disability) and non-clinical outcome measures, i.e. brain MRI, biochemical and hematological safety. The frequent MRI monitoring will allow us to monitor the safety of tolDC in MS patients objectively by measuring T1-enhancing lesions and new and/or enlarging T2 lesions. In this way, unexpected disease activity can be detected timely. The severity of AE will be defined according to the World Health Organization (WHO) toxicity grading scale. The relationship of an AE to the investigational product will be determined by the neurologists and the independent DSMB on the basis of their clinical judgment.

A proportion of the patients may experience MS relapses during the study. A relapse is defined as a new or worsening neurological symptom that occurs in the absence of fever or infection, occurs at least 30 days following the onset of a previous relapse, persists for at least 24 hours and includes an increase in EDSS (Expanded Disability Status Scale) score[41] from the previous assessment matching one of the following: (i) an increase of ≥ 1 on the total scale score; or (ii) an increase of ≥ 2 points on one of the appropriate Functional System Scores (FSS), or (iii) an increase of > 1 point on two or more of the appropriate FSS. Patients will be instructed to notify their neurologist as soon as possible but within 72 hours the latest and will be examined within 7 days of onset of the symptoms. In case of a MS relapse, a course of high-dose steroids can be administered at the discretion of the treating neurologist.

For peer review only - http://bmjopen.bmj.com/site/about/guidelines.xhtml

Study treatment must be discontinued for a given patient if the investigator determines that continuing would result in a significant risk for that patient. In case of terminating the study treatment, patients will still be monitored for safety issues for the duration of the study.

Feasibility

To evaluate the feasibility, successful production of tolDC after leukapheresis will be evaluated. This includes the production of sufficient numbers of tolDC and compliance of the final investigational product to the pre-specified release criteria following cryopreservation.

Secondary outcome measures

Clinical evaluation

The following clinical and laboratory assessments will be performed during a complete physical examination: vital signs, electrocardiogram, routine blood and urine samples, urine pregnancy test in female patients of reproductive potential and serological screening tests, according to the study schedule (Figure 1 and Table 4). Concurrent drug use or new adverse events will be reviewed and recorded.

EDSS is based on a standardized neurological examination and measures impairments in 8 functional systems, including vision, brainstem, pyramidal, cerebellar, sensory, bowel and bladder, mental (cerebral), and ambulation . It has been used for over 20 years as a clinical outcome measure of MS disease progression and consists of a 10-point scale of disease severity ranging from 0, i.e. no disability, to 10, i.e. death from MS[41]. All neurologists involved in patient evaluation will be EDSS training certified.

EDSS will be supplemented by three well-known, quantitative, continuous tests that evaluate ambulation (walking speed by the timed 25 foot walk test or T25FW, arm dexterity

For peer review only

For peer review only - http://bmjopen.bmj.com/site/about/guidelines.xhtml

and function by the 9 Hole Peg Test or 9HPT, and cognition by the Symbol Digit Modalities Test or SDMT).

The effect on disability progression will be characterized by reporting the proportion of patients who are free from disease progression. For this, disability will be assessed based on a sustained clinically relevant change seen in any one of disability assessments: EDSS, T25FW, SDMT or the 9-HTP. Disease progression is defined by: 3-month sustained increase from baseline in the EDSS score (1 point in patients with baseline EDSS score 0 to 5.0; 0.5 point in patients with baseline EDSS score of 5.5 -6.5) or 3-month sustained increase of at least 20% from baseline in the time taken to complete the T25FW or 3-month sustained increase of at least 20% from baseline in the time taken to complete the 9-HPT. Percent change from baseline after treatment in SDMT score will also be recorded.

MRI acquisition and evaluation

MRI is the current gold standard for non-clinical monitoring of MS, and MRI-derived markers have been established as standard outcome measures to monitor the treatment response in various MS clinical trials. MRI will be performed on the same 3T scanner throughout the study. The MRI protocol includes a 3PLANE scout, 3D T1-weighted image pre- and post-gadolinium (Dotarem®, 20 ml) administration (voxel resolution 0.9x0.9x0.9, TR2300.0, TE2.29, TI 900.0), and a 3D FLAIR image (voxel resolution 0.4x0.4x0.9, TR5000.0, TE387.0, TI1800.0). The quantification of number of T2 lesions, T2 lesion load, number and volume of Gd-enhancing lesions, based on 3D FLAIR images and 3D T1-weighted images, is completely automatic avoiding inter- or intra-rater variability. The lesions are quantified within different brain regions. Disease activity or progression on MRI will be evaluated by pre-treatment versus ongoing and post-treatment: (i) change in mean number of enhancing lesions; (ii) change in number of T1 Gd-enhancing and/or new or enlarging T2 lesions; (iii) percent

For peer review only

For peer review only - http://bmjopen.bmj.com/site/about/guidelines.xhtml

change in T2 lesion load; (iv) percent change in brain volume. In order to compare MRI outcome measures from different studies, a standardized acquisition is guaranteed by (i) a uniform MRI protocol, (ii) the use of standard operating procedures for image acquisition and upload, and (iii) training of local MRI operators and study coordinators for acquisition and upload.

Immune-monitoring

To evaluate therapy-related changes in the immune cell profile, peripheral blood (10x10 mL heparin tubes) will be sampled according to the time points depicted in Figure 1 and Table 3, and analyzed by multi-parameter flow cytometry prior to, during and after vaccination. In an attempt to cover the main leukocyte subsets of peripheral blood, the following subsets and their activation status will be enumerated in whole blood samples: $CD4^+$ and $CD8^+$ T cell subpopulations, B cell subsets, natural killer (NK) cells, NKT cells, and myeloid cells. Myelin-specific T cell reactivity will be determined before, during and after completion of administration of tolDC. For this, responsiveness of T cells to myelin antigens will be investigated. As a control, the responsiveness of T cells to unrelated antigens, e.g. cytomegalovirus (CMV) or tetanus toxoid (TT), will be addressed. In doing so, we will be able to assess *ex vivo* the potential risk of inducing opportunistic infections by administering tolDC to MS patients. Finally, patient materials from the studies will be specifically biobanked. We envisage to maximally performing batch measurements and centralized immune monitoring analysis as soon as a dose cohort has reached 3 months follow-up of all patients. For this, extensive immune cell profiling as well as cytokine production of T cells will be analyzed by multi-parameter flow cytometry. Using lineage-specific as well as activation markers, proportions and activation status of the different immune cell subsets will be determined. Besides, antibody titers and memory B cell analysis will be performed.

For peer review only

Patient-reported outcomes

Patient-reported outcomes will be assessed by Multiple Sclerosis Quality of Life-54 (MSQOL-54)[42], a self-reporting questionnaire to detect changes in general and disease-specific quality of life.

Data management and monitoring

Adequate and accurate patient records will be kept enabling the appropriate and required documentation of the study and subsequent verification of the collected data. All data are completed in the electronic case report form (eCRF) for each patient enrolled in this study, including patients who did not start with the investigational treatment. A central data manager affiliated to a clinical research organization (CRO) will perform source data verification. In doing so, study compliance will be monitored, thereby assuring the protection of the rights, safety and well-being of study subjects.

Analysis

Given the design of the study (phase I) and its specific primary end point (safety), no confirmatory statistical testing will be performed. P-values will be calculated but interpreted with caution. Study investigations will be exploratory and conclusions will be based on the complete set of patient evidence.

Analyses of safety variables will be performed with the safety population (all the patients treated with at least one dose of the cell product) by available data only (ADO) analysis. For demographic and safety analysis, i.e. the incidence of adverse events, laboratory values and changes in vital signs, descriptive statistics will be calculated including frequencies for categorical variables and mean and standard deviation, or median and interquartile range for quantitative parameters. In brief, EDSS and other repeatedly measured variables will be

For peer review only

analyzed by means of Mixed Models for Repeated Measurements (MMRM). In case model assumptions are violated (e.g. non-Gaussian errors) or when dealing with ordinal efficacy variables, alternative methods (including non-parametric methods and ordinal regression methods for repeated measurements) will be used. The percentage of patients with 1-point in EDSS improvement will be estimated using a binomial regression model including the treatment and the baseline EDSS. The remainder of variables will be analyzed according to the appropriate statistical test: Chi-square or Fisher's exact test to compare categorical variables, the dependent or independent t-test for continuous Gaussian-distributed variables and the Wilcoxon or Mann-Whitney test for ordinal and non-Gaussian continuous data. The significance level alpha will be set at 0.05 for two-tailed analysis. Variables to assess the analysis of secondary and tertiary outcome measures will be performed with per protocol population. Continuous variables as a minimum will be described by number of total and non-missing observations (n) and the appropriate location-scale statistics including arithmetic mean, standard deviation (SD), minimum, median, Q1-Q3, and maximum. Categorical variables will be presented using the number of non-missing observations (n) or the number of patients in the population (N) as applicable and percentages (%). Two-sided 95% (exact) Confidence Intervals (CI) will be provided when relevant.

For peer review only

Ethics and dissemination

Institutional Review Board approval

The study protocol was approved by the Federal Agency for Medicines and Health Products (FAMHP, Belgium), the Spanish Agency of Medicines and Medical Devices (Spain), the Ethics Committee of the Antwerp University Hospital (Belgium), and the Ethics Committees of Hospital Germans Trias i Pujol and Clínica Universidad de Navarra.

For peer review only - http://bmjopen.bmj.com/site/about/guidelines.xhtml

Patient protection

The study will be conducted in agreement with either the Declaration of Helsinki or the laws and regulations of the country, whichever provides the greatest protection of the patient. In the Belgian context, the Law of May 7, 2004 ("Wet van 7 mei 2004 inzake experimenten op de menselijke persoon") applies. The study will be conducted in agreement with the ICH Harmonized Tripartite Guideline for Good Clinical Practice.

Informed consent

All patients will be informed of the aim of the study, the possible adverse events, the procedures and possible hazards to which he/she will be exposed. They will be informed as to the strict confidentiality of their patient data, but that their medical records may be reviewed for trial purposes by authorized individuals other than their treating physician.

For peer review only

Dissemination

The results of the clinical trials will be published in a peer-reviewed journal. In addition, the results will be presented at scientific conferences and to patient associations. In particular for Spanish patient associations, dissemination of results will be handled by the Spanish Agency of Medicines and Medical Devices whose content is written in layman's terms. On completion of the trial and after publication of the study results, data requests can be submitted to the researchers.

For peer review only

For peer review only - http://bmjopen.bmj.com/site/about/guidelines.xhtml

DISCUSSION

Although the first phase I clinical trials have demonstrated promising results with regard to safety of administering tolDC in other autoimmune diseases, numerous questions remain concerning which dose, treatment schedule or route of administration is best with regard to safety, efficacy and related costs of treatment with tolDC. In this collaborative effort, each patient will receive 6 repetitive injections of 5, 10 or 15 x 10^6 autologous myelin-derived peptide mix-loaded tolDC, intradermal or intranodal, i.e. 4 administrations once every 2 weeks and 2 administrations once every 4 weeks. Previously, others evaluated the safety of intravenous administration of tolerogenic DC in MS and neuromyelitis optica (NMO) patients (table 1)[43]. In the current study, harmonization of clinical, MRI and immunological evaluations of the patients will enable us to compare results between two phase I clinical trials evaluating the safety and feasibility of autologous tolDC administration in patients with active MS. To our knowledge, this will be the first time that different routes of administration are set side by side for a cell therapy product.

In conclusion, our protocols envisage to restore tolerance to predefined myelin-peptide antigens using peptide-loaded tolDC. From the results of these two phase I clinical studies, the optimal dose and administration route will be selected for future phase II trials investigating the efficacy of this patient-tailored treatment in MS.

For peer review only - http://bmjopen.bmj.com/site/about/guidelines.xhtml

Acknowledgements

We thank all the patients that contributed to the preclinical research by donating samples. Our gratitude goes to Marleen Breuls, MS nurse and to the study nurses of the Neurology Department of the Antwerp University Hospital, Linda Wagemaekers, Caroline Vinck and Maren Wyckmans as well as Sandra Vidal, study nurse at Clinical Trial Unit in Research Foundation Germans Trias i Pujol-IGTP. We thank the Spanish Clinical Research Network-SCReN for the support on Pharmacovigilance and Case Report Form implementation for the study. We thank also Jorge Luis Reverter, at Endocrinology Department at Hospital Germans Trias i Pujol for the treatment administration. We thank the patients and Belgian and Spanish MS patient organizations for their participation in the stakeholder committee. We thank the scientific advisory board and the data safety monitoring board members for their contributions.

For peer review only

Author contributions

BW, ZB, CRT, EMC, WVH and NC conceived and designed the study. WPL, GN, MDL, GA, AR, MC, IW and NC participated in logistical planning of the study. BW and SPR wrote the initial draft of the manuscript. BW, SPR, CRT, MJM, IW, EMC and NC wrote and reviewed the manuscript. NH provided the statistical support for the sample size estimates and the design of the statistical analysis. BW, SPR, MJM, JD, WPL, GN, MDL, IW, PC, PMP, WVH, AR, TB, GA, MC, JNB, ATS, BQS, ALDC, SI, FP, AK, HV, CCG, HW, SMVH, ATB, AMB, AMV, NH, ZB, EMC, NC and CRT made significant contributions to the development and conceptualization of the protocol. BW, SPR, MJM, JD, WPL, GN, MDL, IW, PC, PMP, WVH, AR, TB, GA, MC, JNB, ATS, BQS, ALDC, SI, FP, AK, HV, CCG, HW, SMVH, ATB, AMB, AMV, NH, ZB, EMC, NC and CRT reviewed the draft versions of this paper and have read and approved the final manuscript.

For peer review only

For peer review only - http://bmjopen.bmj.com/site/about/guidelines.xhtml

463 **Funding**

464 This work was supported by positive discussion through the A FACTT network (Cost Action
465 BM1305: www.afactt.eu). COST is supported by the EU Framework Program Horizon 2020.
466 This RESTORE project has received funding from the European Union's Horizon 2020
467 research and innovation program under grant agreement number 779316. Further support
468 was provided by an applied biomedical research project of the Institute for the Promotion of
469 Innovation by Science and Technology in Flanders (IWT-TBM 140191), by projects
470 PI11/02416, PI14/01175,PI16/01737 and PT13/0002/0038 (Platform for Clinical Research
471 and Clinical Trial Units, Spanish Clinical Research Network, SCReN), integrated in the Plan
472 Nacional de I+D+I and co-supported by the Health Institute Carlos III - Subdirección General
473 de Evaluación y Fomento de la Investigación of the Spanish Ministry of Economy and
474 Competitiveness and the Fondo Europeo de Desarrollo Regional (FEDER), and project
475 07/2410 Fundació La Marato de TV3. Furthermore, the authors received research funding
476 from Sanofi Genzyme, Belgium. Judith Derdelinckx holds a PhD fellowship from the Research
477 Foundation Flanders (FWO). Dr. Presas-Rodríguez is a neurologist who has received a grant
478 of Hospital Germans Trias i Pujol ("Germans Trias Talents 2016-2018") to work on this
479 project. Dr. Willekens is a neurologist at the Antwerp University Hospital supported by a
480 research fellowship (2016-2018) of the University of Antwerp to work on this project and she
481 currently holds a clinical PhD fellowship from the Research Foundation Flanders (FWO
482 1701919N). Spanish Patient association "Treball de Vida" (Associació d'Afectats d'Esclerosi
483 Múltiple del Barcelonès Nord i Maresme) and patient Ana Mª Calvo Marsal have donated
484 funding to the Hospital Germans Trias i Pujol MS Unit.

485

486

For peer review only - http://bmjopen.bmj.com/site/about/guidelines.xhtml

Conflict of interest

CCG received speaker honoraria and travel expenses for attending meeting from Genzyme, Novartis Pharma GmbH, and Bayer Health Care. Her work is funded by the German Ministry for Education and Research (BMBF; 01GI1603A) and the German Research Foundation (DFG; GR3946/3-1 and SFB128 A09).

HW receives honoraria for acting as a member of Scientific Advisory Boards and as consultant for Biogen, Evgen, MedDay Pharmaceuticals, Merck Serono, Novartis, Roche Pharma AG, Sanofi-Genzyme, as well as speaker honoraria and travel support from Alexion, Biogen, Cognomed, F. Hoffmann-La Roche Ltd., Gemeinnützige Hertie-Stiftung, Merck Serono, Novartis, Roche Pharma AG, Sanofi-Genzyme, TEVA, and WebMD Global. Prof. Wiendl is acting as a paid consultant for Abbvie, Actelion, Biogen, IGES, Novartis, Roche, Sanofi-Genzyme, and the Swiss Multiple Sclerosis Society. His research is funded by the German Ministry for Education and Research (BMBF; 01FI1601E, 01GI1603A, and O1GI1603D), Deutsche Forschungsgesellschaft (DFG; SFB128 A09, A10, Z02, V and SFB1009 A03), Else Kröner Fresenius Foundation, Fresenius Foundation, Hertie Foundation, NRW Ministry of Education and Research, Interdisciplinary Center for Clinical Studies (IZKF) Muenster and RE Children’s Foundation, Biogen GmbH, GlaxoSmithKline GmbH, Roche Pharma AG, Sanofi-Genzyme.

The institution of BW receives honoraria for acting as a member of Scientific Advisory Boards for Biogen, Merck Serono, Roche, Sanofi-Genzyme, Novartis and speaker honoraria and travel support from Biogen, Merck Serono, Roche, Sanofi-Genzyme, Novartis, TEVA.

CRT receives honoraria for acting as a member of Scientific Advisory Boards for Biogen, and Merck Serono, and speaker honoraria or travel support from Biogen, Merck Serono, Roche, Sanofi-Genzyme and Novartis.

For peer review only

SPR receives speaker honoraria or travel support from Biogen, Merck Serono, Roche, Sanofi-Genzyme and Novartis.

The other authors report no conflict of interest.

For peer review only

Table 1 Overview of clinical trials using tolDC as therapeutic intervention in autoimmune diseases

Reference	Indication	Study design	Number of patients	Cell product and control condition	Dose	Administration mode	Primary outcome measure	Results	Immunological effects
Zubizarreta I. et al. 2019[44]	MS and NMO	open-label, dose-escalation, phase Ib	8 MS and 4 NMO	autologous tolDC loaded with either myelin peptides or AQP4	50 × 10^6, 100 × 10^6, 150 × 10^6, and 300 × 10^6 tolDC in total, separated in three independent doses administered every 2 weeks	intravenous	safety and tolerability	well tolerated no serious adverse events	↑ IL-10 production in peptide-stimulated PBMCs and ↑in the frequency of Tr1 (a regulatory T cell)
Bell GM et al. 2017[28]	Inflammatory arthritis	unblinded, dose-escalation, randomized, phase I	9	autologous tolDC loaded with autologous synovial fluid as a source of autoantigens	1×10^6, 3×10^6 or 10×10^6 tolDC arthroscopically vs saline only	intra-articular	flare of disease in the target knee within 5 days of treatment	no target knee flares within 5 days of treatment	no consistent immunomodulatory effects in peripheral blood
Benham H. et al. 2015[27]	Rheumatoid arthritis	open-label, controlled, phase I	34	autologous DCs modified with a nuclear factor kappaB (NF-kappaB) inhibitor exposed to four citrullinated peptide antigens, designated "Rheumavax,"	a low dose of 1 × 106 DCs and a high dose of 5 × 106	intradermal	safety	mild adverse events	↑ in effector T cells and an ↑ ratio of regulatory to effector T cells; ↓ in serum interleukin-15 (IL-15), IL-29, CX3CL1, and CXCL11; ↓ T cell IL-6 responses to vimentin (447-455)-Cit450 relative to controls
Jauregui-Amezaga A. et al. 2015[29]	Crohn's disease	open-label, dose-escalation, phase I	9	autologous tolDC	first three cohorts: a single injection of 2 x 10^6, 5 x 10^6 or 10 x 10^6 tolDC; last three cohorts: 3 bi-weekly injections (same dose escalation schedule)	intraperitoneal	safety	no adverse effects	
Giannoukakis N.et al. 2011[26]	Diabetes type 1	randomized, double-blind, phase I	10	autologous unmanipulated dendritic cells or tolDC	10 x 10^6 cells once every 2 weeks for a total of four administrations	intradermal	safety	no adverse effects	↑ in the frequency of peripheral B220+ CD11c- B cells

Abbreviations: MS= Multiple Sclerosis, NMO= Neuromyelitis Optica, tolDC= tolerogenic dendritic cells, PBMC=Peripheral Blood Mononuclear Cell, IL-10= Interleukin-10, Tr1= regulatory T-cell type

For peer review only - http://bmjopen.bmj.com/site/about/guidelines.xhtml

543 **Table 2: In- and exclusion criteria.** All the inclusion criteria must be fulfilled. The presence of
544 any of the exclusion criteria shall exclude the patient from the study.

**Inclusion criteria**	**Exclusion criteria**
MS according to most recent McDonald criteria	Previous use of immunosuppressive or cytostatic treatment, including mitoxantrone, cladribine, alemtuzumab or bone marrow transplantation or stem cell transplantation at any time prior to enrolment
Age 18-60 years	Treatment with fingolimod or natalizumab or dimethylfumarate in the past 12 weeks or teriflunomide within the past 15 weeks or ocrelizumab/rituximab within the past 6 months prior to the first administration
EDSS of 0-6.5 inclusive	Pregnancy or planning pregnancy in the next 12 months and breast feeding
First signs or symptoms at least 3 months prior to enrolment in the study	Drug or alcohol abuse

For peer review only

Study protocol for tolDC administration in patients with active MS

Active MS (relapsing and/or progressive): 1 relapse in the past year and/or at least 1 enhancing lesion on brain MRI in the past year; at least 1 new or enlarging T2 lesion in comparison with a reference scan from maximum 1 year before	Inability to undergo MRI assessments
Normal peripheral B-cell count after treatment with ocrelizumab	
No evidence of relapse for at least 30 days prior to start of screening and throughout during the screening phase	History of or actual signs of immunodeficiency or malignancies
Positive T cell reactivity response to a mix of 7 myelin-derived peptides	Concurrent clinically relevant cardiac, immunological, pulmonary, neurological, renal or other major disease
Able to sign informed consent and comply with the protocol assessments	Active or chronic infection (Hepatitis B or C, HIV, syphilis or tuberculosis)
No wish to be treated with currently available DMT	Splenectomy
Appropriate venous access and *adequate cervical lymph nodes on ultrasound	

For peer review only

mapping	
Use of adequate contraceptive measures. Women of childbearing potential can only be included in the study following use of adequate contraceptive measures. Accepted methods of contraception include use of hormonal contraceptives (oral, intravaginal, intrauterine, or transdermal), intrauterine devices, sterilization or postmenopausal status, use of condoms with spermicide.	

*only in TOLERVIT-MS

For peer review only

TABLE 3: Outline of the cell doses and patient numbers, per phase I clinical trial, in the dose escalation cohorts for intradermal and intranodal administration of tolDC. Abbreviations used: i.d., intradermal; tolDC, tolerogenic dendritic cells; N, number.

Cohort	Treatment Regimen	Patient numbers
**1**	6 i.d./i.n. injections of $5x10^6$ tolDC	N=3 (+1+1)
**2**	6 i.d./i.n. injections of $10x10^6$ tolDC	N=3 (+1+1)
**3**	6 i.d./i.n. injections of $15x10^6$ tolDC	N=3 (+1+1)

For peer review only

Study protocol for tolDC administration in patients with active MS

TABLE 4: Study calendar. The different visits, examinations, tests and the tolDC administration are detailed on the tabular study schedule overview.

	Screening		Treatment period							Safety follow up			
	**Ts**	**Baseline (T0)**	**V1**	**V1 + 1day**	**V2**	**V3**	**V4**	**V5**	**V6**	**F1**	**F3**	**F6**	**F12**
**Informed consent**	•												
**In- and exclusion criteria**	•												
**IFN-γ EliSPOT (T cell reactivity assay)**	•		•				•+ 1w		•+ 1w		•		
**Leukapheresis**		•											
**TolDC preparation**		•											
**TolDC treatment**			•		•	•	•	•	•				
**Patient evaluation:**													
- **Vital signs (HR, BP,…)**	•	•	•	•	•	•	•	•	•	•	•	•	•
- **ECG**	•	•	•	•	•	•	•	•	•	•	•	•	•
- **Blood analysis for safety (1x5ml EDTA tube and 2x10ml serum tubes)**	•	•	•	•	•	•	•	•	•	•	•	•	•
- **Urine pregnancy test**	•	•	•		•	•	•	•	•	•	•	•	•
**Full neurological examination (EDSS, 9-HPT, T25FW, SDMT, MSQOL-54)**	•		•				•		•	•	•	•	•
**Pain score after treatment injection**			•		•	•	•	•	•				
**MRI**	•	•	•		•		•+ 1w	•+ 1w	•+ 1w		•	•	•
**Immunomonitoring (10x10ml heparin tubes + 1 serum tube)**			•				•+ 1w		•+ 1w		•		
**Biobanking for follow up immunomonitoring**										•		•	•
**(Serious) Adverse events & concomitant medication**	**Continuous**												

For peer review only

Study protocol for tolDC administration in patients with active MS

For peer review only

For peer review only - http://bmjopen.bmj.com/site/about/guidelines.xhtml

References

1. Lublin FD, Reingold SC, Cohen JA, et al. Defining the clinical course of multiple sclerosis: the 2013 revisions. *Neurology* 2014;83(3):278-86. doi: 10.1212/wnl.0000000000000560 [published Online First: 2014/05/30]
2. Scalfari A, Neuhaus A, Daumer M, et al. Onset of secondary progressive phase and long-term evolution of multiple sclerosis. *Journal of neurology, neurosurgery, and psychiatry* 2014;85(1):67-75. doi: 10.1136/jnnp-2012-304333 [published Online First: 2013/03/15]
3. Cree BA, Gourraud PA, Oksenberg JR, et al. Long-term evolution of multiple sclerosis disability in the treatment era. *Annals of neurology* 2016;80(4):499-510. doi: 10.1002/ana.24747 [published Online First: 2016/07/28]
4. Scolding N, Barnes D, Cader S, et al. Association of British Neurologists: revised (2015) guidelines for prescribing disease-modifying treatments in multiple sclerosis. *Practical neurology* 2015;15(4):273-9. doi: 10.1136/practneurol-2015-001139 [published Online First: 2015/06/24]
5. Dendrou CA, Fugger L, Friese MA. Immunopathology of multiple sclerosis. *Nature reviews Immunology* 2015;15(9):545-58. doi: 10.1038/nri3871 [published Online First: 2015/08/08]
6. Grigoriadis N, van Pesch V. A basic overview of multiple sclerosis immunopathology. *Eur J Neurol* 2015;22 Suppl 2:3-13. doi: 10.1111/ene.12798 [published Online First: 2015/09/17]
7. Bielekova B, Goodwin B, Richert N, et al. Encephalitogenic potential of the myelin basic protein peptide (amino acids 83-99) in multiple sclerosis: results of a phase II clinical trial with an altered peptide ligand. *Nature medicine* 2000;6(10):1167-75. doi: 10.1038/80516 [published Online First: 2000/10/04]
8. Bielekova B, Sung MH, Kadom N, et al. Expansion and functional relevance of high-avidity myelin-specific CD4+ T cells in multiple sclerosis. *Journal of immunology (Baltimore, Md : 1950)* 2004;172(6):3893-904. [published Online First: 2004/03/09]
9. Wallstrom E, Khademi M, Andersson M, et al. Increased reactivity to myelin oligodendrocyte glycoprotein peptides and epitope mapping in HLA DR2(15)+ multiple sclerosis. *European journal of immunology* 1998;28(10):3329-35. doi: 10.1002/(sici)1521-4141(199810)28:10<3329::aid-immu3329>3.0.co;2-b [published Online First: 1998/11/10]
10. Grau-Lopez L, Raich D, Ramo-Tello C, et al. Specific T-cell proliferation to myelin peptides in relapsing-remitting multiple sclerosis. *Eur J Neurol* 2011;18(8):1101-4. doi: 10.1111/j.1468-1331.2010.03307.x [published Online First: 2011/07/14]
11. Van Brussel I, Lee WP, Rombouts M, et al. Tolerogenic dendritic cell vaccines to treat autoimmune diseases: can the unattainable dream turn into reality? *Autoimmunity reviews* 2014;13(2):138-50. doi: 10.1016/j.autrev.2013.09.008 [published Online First: 2013/10/15]
12. Willekens B, Cools N. Beyond the Magic Bullet: Current Progress of Therapeutic Vaccination in Multiple Sclerosis. *CNS drugs* 2018 doi: 10.1007/s40263-018-0518-4 [published Online First: 2018/05/16]
13. Gonsette RE. Self-tolerance in multiple sclerosis. *Acta Neurol Belg* 2012;112(2):133-40. doi: 10.1007/s13760-012-0061-x [published Online First: 2012/03/28]
14. Lutterotti A, Martin R. Antigen-specific tolerization approaches in multiple sclerosis. *Expert opinion on investigational drugs* 2014;23(1):9-20. doi: 10.1517/13543784.2014.844788 [published Online First: 2013/10/25]
15. Gross CC, Jonuleit H, Wiendl H. Fulfilling the dream: tolerogenic dendritic cells to treat multiple sclerosis. *European journal of immunology* 2012;42(3):569-72. doi: 10.1002/eji.201242402 [published Online First: 2012/04/11]
16. Berneman Z, Lee W, Willekens B, et al. Towards the Clinical Use of Tolerogenic Dendritic Cells in Multiple Sclerosis by Applying the Immunomodulatory Effects of 1, 25-dihydroxyvitamin D3 on Dendritic Cells to Promote Induction of T Cell Hyporesponsiveness to Myelin-Derived Antigens. *Cytotherapy* 2016;18(6):S95-S96.

For peer review only

For peer review only - http://bmjopen.bmj.com/site/about/guidelines.xhtml

17. Using cells to cure disease: Tolerogenic dendritic cells for the treatment of MS. MULTIPLE SCLEROSIS JOURNAL; 2016. SAGE PUBLICATIONS LTD 1 OLIVERS YARD, 55 CITY ROAD, LONDON EC1Y 1SP, ENGLAND.
18. Ten Brinke A, Hilkens CM, Cools N, et al. Clinical Use of Tolerogenic Dendritic Cells-Harmonization Approach in European Collaborative Effort. *Mediators of inflammation* 2015;2015:471719. doi: 10.1155/2015/471719 [published Online First: 2016/01/29]
19. Nuyts AH, Lee WP, Bashir-Dar R, et al. Dendritic cells in multiple sclerosis: key players in the immunopathogenesis, key players for new cellular immunotherapies? *Multiple sclerosis (Houndmills, Basingstoke, England)* 2013;19(8):995-1002. doi: 10.1177/1352458512473189 [published Online First: 2013/02/02]
20. Florez-Grau G, Zubizarreta I, Cabezon R, et al. Tolerogenic Dendritic Cells as a Promising Antigen-Specific Therapy in the Treatment of Multiple Sclerosis and Neuromyelitis Optica From Preclinical to Clinical Trials. *Frontiers in immunology* 2018;9:1169. doi: 10.3389/fimmu.2018.01169 [published Online First: 2018/06/16]
21. Lee W-P, Willekens B, Cras P, et al. Immunomodulatory Effects of 1, 25-Dihydroxyvitamin D3 on Dendritic Cells Promote Induction of T Cell Hyporesponsiveness to Myelin-Derived Antigens. *Journal of Immunology Research* 2016;2016
22. Raich-Regue D, Grau-Lopez L, Naranjo-Gomez M, et al. Stable antigen-specific T-cell hyporesponsiveness induced by tolerogenic dendritic cells from multiple sclerosis patients. *European journal of immunology* 2012;42(3):771-82. doi: 10.1002/eji.201141835 [published Online First: 2012/04/11]
23. Naranjo-Gomez M, Raich-Regue D, Onate C, et al. Comparative study of clinical grade human tolerogenic dendritic cells. *Journal of translational medicine* 2011;9:89. doi: 10.1186/1479-5876-9-89 [published Online First: 2011/06/11]
24. Mansilla MJ, Selles-Moreno C, Fabregas-Puig S, et al. Beneficial effect of tolerogenic dendritic cells pulsed with MOG autoantigen in experimental autoimmune encephalomyelitis. *CNS neuroscience & therapeutics* 2015;21(3):222-30. doi: 10.1111/cns.12342 [published Online First: 2014/11/19]
25. Mansilla MJ, Contreras-Cardone R, Navarro-Barriuso J, et al. Cryopreserved vitamin D3-tolerogenic dendritic cells pulsed with autoantigens as a potential therapy for multiple sclerosis patients. *Journal of neuroinflammation* 2016;13(1):113. doi: 10.1186/s12974-016-0584-9 [published Online First: 2016/05/22]
26. Giannoukakis N, Phillips B, Finegold D, et al. Phase I (safety) study of autologous tolerogenic dendritic cells in type 1 diabetic patients. *Diabetes care* 2011;34(9):2026-32. doi: 10.2337/dc11-0472 [published Online First: 2011/06/18]
27. Benham H, Nel HJ, Law SC, et al. Citrullinated peptide dendritic cell immunotherapy in HLA risk genotype-positive rheumatoid arthritis patients. *Science translational medicine* 2015;7(290):290ra87. doi: 10.1126/scitranslmed.aaa9301 [published Online First: 2015/06/05]
28. Bell GM, Anderson AE, Diboll J, et al. Autologous tolerogenic dendritic cells for rheumatoid and inflammatory arthritis. *Annals of the rheumatic diseases* 2017;76(1):227-34. doi: 10.1136/annrheumdis-2015-208456 [published Online First: 2016/04/28]
29. Jauregui-Amezaga A, Cabezon R, Ramirez-Morros A, et al. Intraperitoneal Administration of Autologous Tolerogenic Dendritic Cells for Refractory Crohn's Disease: A Phase I Study. *Journal of Crohn's & colitis* 2015;9(12):1071-8. doi: 10.1093/ecco-jcc/jjv144 [published Online First: 2015/08/26]
30. De Vries IJ, Krooshoop DJ, Scharenborg NM, et al. Effective migration of antigen-pulsed dendritic cells to lymph nodes in melanoma patients is determined by their maturation state. *Cancer research* 2003;63(1):12-7. [published Online First: 2003/01/09]
31. Ridolfi R, Riccobon A, Galassi R, et al. Evaluation of in vivo labelled dendritic cell migration in cancer patients. *Journal of translational medicine* 2004;2(1):27. doi: 10.1186/1479-5876-2-27 [published Online First: 2004/08/03]

For peer review only - http://bmjopen.bmj.com/site/about/guidelines.xhtml

32. Morse MA, Coleman RE, Akabani G, et al. Migration of human dendritic cells after injection in patients with metastatic malignancies. *Cancer research* 1999;59(1):56-8. [published Online First: 1999/01/19]
33. Lesterhuis WJ, de Vries IJ, Schreibelt G, et al. Route of administration modulates the induction of dendritic cell vaccine-induced antigen-specific T cells in advanced melanoma patients. *Clinical cancer research : an official journal of the American Association for Cancer Research* 2011;17(17):5725-35. doi: 10.1158/1078-0432.ccr-11-1261 [published Online First: 2011/07/21]
34. de Vries IJ, Lesterhuis WJ, Barentsz JO, et al. Magnetic resonance tracking of dendritic cells in melanoma patients for monitoring of cellular therapy. *Nature biotechnology* 2005;23(11):1407-13. doi: 10.1038/nbt1154 [published Online First: 2005/11/01]
35. Sun BL, Wang LH, Yang T, et al. Lymphatic drainage system of the brain: A novel target for intervention of neurological diseases. *Progress in neurobiology* 2018;163-164:118-43. doi: 10.1016/j.pneurobio.2017.08.007 [published Online First: 2017/09/14]
36. Louveau A, Smirnov I, Keyes TJ, et al. Structural and functional features of central nervous system lymphatic vessels. *Nature* 2015;523(7560):337-41. doi: 10.1038/nature14432 [published Online First: 2015/06/02]
37. Rieckmann P, Centonze D, Elovaara I, et al. Unmet needs, burden of treatment, and patient engagement in multiple sclerosis: A combined perspective from the MS in the 21st Century Steering Group. *Multiple sclerosis and related disorders* 2018;19:153-60. doi: 10.1016/j.msard.2017.11.013 [published Online First: 2017/12/15]
38. Polman CH, Reingold SC, Banwell B, et al. Diagnostic criteria for multiple sclerosis: 2010 revisions to the McDonald criteria. *Annals of neurology* 2011;69(2):292-302. doi: 10.1002/ana.22366 [published Online First: 2011/03/10]
39. Thompson AJ, Banwell BL, Barkhof F, et al. Diagnosis of multiple sclerosis: 2017 revisions of the McDonald criteria. *The Lancet Neurology* 2018;17(2):162-73. doi: 10.1016/s1474-4422(17)30470-2 [published Online First: 2017/12/26]
40. Le Tourneau C, Lee JJ, Siu LL. Dose escalation methods in phase I cancer clinical trials. *Journal of the National Cancer Institute* 2009;101(10):708-20. doi: 10.1093/jnci/djp079 [published Online First: 2009/05/14]
41. Kurtzke JF. Rating neurologic impairment in multiple sclerosis: an expanded disability status scale (EDSS). *Neurology* 1983;33(11):1444-52. [published Online First: 1983/11/01]
42. Vickrey BG, Hays RD, Harooni R, et al. A health-related quality of life measure for multiple sclerosis. *Quality of life research : an international journal of quality of life aspects of treatment, care and rehabilitation* 1995;4(3):187-206. [published Online First: 1995/06/01]
43. Nafarrate IZ, Florez G, Vila G, et al. Phase 1b clinical trial with antigen-specific tolerogenic dendritic in Multiple Sclerosis and Neuromyelitis optica: safety and immunological effects (P2.330). *Neurology* 2017;88(16 Supplement):P2.330.
44. Zubizarreta I, Florez-Grau G, Vila G, et al. Immune tolerance in multiple sclerosis and neuromyelitis optica with peptide-loaded tolerogenic dendritic cells in a phase 1b trial. *Proceedings of the National Academy of Sciences of the United States of America* 2019;116(17):8463-70. doi: 10.1073/pnas.1820039116 [published Online First: 2019/04/10]

For peer review only - http://bmjopen.bmj.com/site/about/guidelines.xhtml

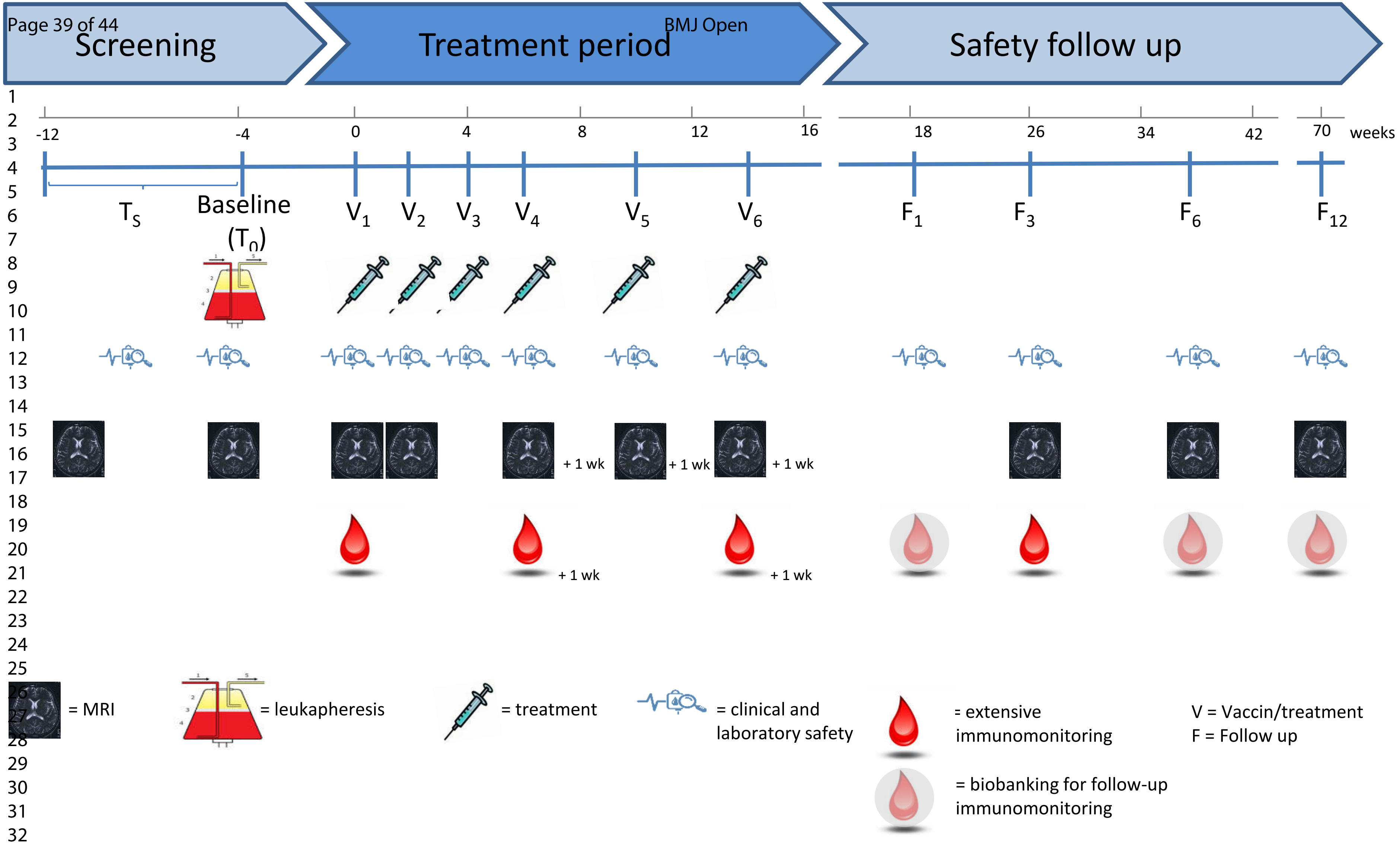

Figure 1: Study design. Treatment will start at V_1, approximately 4 weeks after the leukapheresis. Patients will receive additional injections on week +2 (V_2), week +4 (V_3), week +6 (V_4), week +10 (V_5) and week +14 (V_6). Patients will have follow up visits one month, and 3, 6 and 12 months following the last study treatment.

		Page number	Line numbers
**Administrative information**			
Title	1	1	1, 2, 3
Trial registration	2a	3	69-71
	2b		
Protocol version	3		
Funding	4	24	461-482
Roles and responsibilities	5a	23	453.454
	5b		
	5c		
	5d		
**Introduction**			
Background and rationale	6a	5,6,7,8	86-181
	6b		
Objectives	7	9	189-201
Trial design	8	9	183-188
**Methods: Participants, interventions, and outcomes**			
Study setting	9	9	185-186
Eligibility criteria	10	10	215-219
Interventions	11a	12	251-262
	11b	10.11	221.223
	11c		
	11d		
Outcomes	12	13-17	264-359
Participant timeline	13	32	table 4, figure 1
Sample size	14	10.11	220-233
Recruitment	15	9, 10	185,186, 216-219
Methods: Assignment of interventions (for controlled trials)			
	16-17	not applicable, open-label study	
Methods: Data collection, management, and analysis			
Data collection methods	18a	17	360-367
	18b		
Data management	19		
Statistical methods	20a	17-18	368-395
	20b		
	20c		
Methods: monitoring			
Data monitoring	21a	11	231
	21b	17	360-367
Harms	22	13.17	272-274,360-367
Auditing	23		
Ethics and dissemination			
Research ethics approval	24	18	397-401
Protocol amendments	25		
Consent or assent	26a	19	408-412
	26b		
Confidentiality	27		
Declaration of interests	28	25.26	485-511
Access to data	29		
Ancillary and post-trial care	30		
Dissemination policy	31a	20	414-420
	31b		

For peer review only

	31c		
Appendices			
Informed consent materials	32		
Biological specimens	33		

For peer review only

SPIRIT

STANDARD PROTOCOL ITEMS: RECOMMENDATIONS FOR INTERVENTIONAL TRIALS

SPIRIT 2013 Checklist: Recommended items to address in a clinical trial protocol and related documents*

Section/item	Item No	Description
**Administrative information**		
Title	1	Descriptive title identifying the study design, population, interventions, and, if applicable, trial acronym
Trial registration	2a	Trial identifier and registry name. If not yet registered, name of intended registry
	2b	All items from the World Health Organization Trial Registration Data Set
Protocol version	3	Date and version identifier
Funding	4	Sources and types of financial, material, and other support
Roles and responsibilities	5a	Names, affiliations, and roles of protocol contributors
	5b	Name and contact information for the trial sponsor
	5c	Role of study sponsor and funders, if any, in study design; collection, management, analysis, and interpretation of data; writing of the report; and the decision to submit the report for publication, including whether they will have ultimate authority over any of these activities
	5d	Composition, roles, and responsibilities of the coordinating centre, steering committee, endpoint adjudication committee, data management team, and other individuals or groups overseeing the trial, if applicable (see Item 21a for data monitoring committee)
**Introduction**		
Background and rationale	6a	Description of research question and justification for undertaking the trial, including summary of relevant studies (published and unpublished) examining benefits and harms for each intervention
	6b	Explanation for choice of comparators
Objectives	7	Specific objectives or hypotheses

For peer review only

Trial design	8	Description of trial design including type of trial (eg, parallel group, crossover, factorial, single group), allocation ratio, and framework (eg, superiority, equivalence, noninferiority, exploratory)

Methods: Participants, interventions, and outcomes

Study setting	9	Description of study settings (eg, community clinic, academic hospital) and list of countries where data will be collected. Reference to where list of study sites can be obtained
Eligibility criteria	10	Inclusion and exclusion criteria for participants. If applicable, eligibility criteria for study centres and individuals who will perform the interventions (eg, surgeons, psychotherapists)
Interventions	11a	Interventions for each group with sufficient detail to allow replication, including how and when they will be administered
	11b	Criteria for discontinuing or modifying allocated interventions for a given trial participant (eg, drug dose change in response to harms, participant request, or improving/worsening disease)
	11c	Strategies to improve adherence to intervention protocols, and any procedures for monitoring adherence (eg, drug tablet return, laboratory tests)
	11d	Relevant concomitant care and interventions that are permitted or prohibited during the trial
Outcomes	12	Primary, secondary, and other outcomes, including the specific measurement variable (eg, systolic blood pressure), analysis metric (eg, change from baseline, final value, time to event), method of aggregation (eg, median, proportion), and time point for each outcome. Explanation of the clinical relevance of chosen efficacy and harm outcomes is strongly recommended
Participant timeline	13	Time schedule of enrolment, interventions (including any run-ins and washouts), assessments, and visits for participants. A schematic diagram is highly recommended (see Figure)
Sample size	14	Estimated number of participants needed to achieve study objectives and how it was determined, including clinical and statistical assumptions supporting any sample size calculations
Recruitment	15	Strategies for achieving adequate participant enrolment to reach target sample size

Methods: Assignment of interventions (for controlled trials)

Allocation:

For peer review only - http://bmjopen.bmj.com/site/about/guidelines.xhtml

Sequence generation	16a	Method of generating the allocation sequence (eg, computer-generated random numbers), and list of any factors for stratification. To reduce predictability of a random sequence, details of any planned restriction (eg, blocking) should be provided in a separate document that is unavailable to those who enrol participants or assign interventions
Allocation concealment mechanism	16b	Mechanism of implementing the allocation sequence (eg, central telephone; sequentially numbered, opaque, sealed envelopes), describing any steps to conceal the sequence until interventions are assigned
Implementation	16c	Who will generate the allocation sequence, who will enrol participants, and who will assign participants to interventions
Blinding (masking)	17a	Who will be blinded after assignment to interventions (eg, trial participants, care providers, outcome assessors, data analysts), and how
	17b	If blinded, circumstances under which unblinding is permissible, and procedure for revealing a participant’s allocated intervention during the trial
**Methods: Data collection, management, and analysis**		
Data collection methods	18a	Plans for assessment and collection of outcome, baseline, and other trial data, including any related processes to promote data quality (eg, duplicate measurements, training of assessors) and a description of study instruments (eg, questionnaires, laboratory tests) along with their reliability and validity, if known. Reference to where data collection forms can be found, if not in the protocol
	18b	Plans to promote participant retention and complete follow-up, including list of any outcome data to be collected for participants who discontinue or deviate from intervention protocols
Data management	19	Plans for data entry, coding, security, and storage, including any related processes to promote data quality (eg, double data entry; range checks for data values). Reference to where details of data management procedures can be found, if not in the protocol
Statistical methods	20a	Statistical methods for analysing primary and secondary outcomes. Reference to where other details of the statistical analysis plan can be found, if not in the protocol
	20b	Methods for any additional analyses (eg, subgroup and adjusted analyses)
	20c	Definition of analysis population relating to protocol non-adherence (eg, as randomised analysis), and any statistical methods to handle missing data (eg, multiple imputation)

Methods: Monitoring

Data monitoring	21a	Composition of data monitoring committee (DMC); summary of its role and reporting structure; statement of whether it is independent from the sponsor and competing interests; and reference to where further details about its charter can be found, if not in the protocol. Alternatively, an explanation of why a DMC is not needed
	21b	Description of any interim analyses and stopping guidelines, including who will have access to these interim results and make the final decision to terminate the trial
Harms	22	Plans for collecting, assessing, reporting, and managing solicited and spontaneously reported adverse events and other unintended effects of trial interventions or trial conduct
Auditing	23	Frequency and procedures for auditing trial conduct, if any, and whether the process will be independent from investigators and the sponsor

Ethics and dissemination

Research ethics approval	24	Plans for seeking research ethics committee/institutional review board (REC/IRB) approval
Protocol amendments	25	Plans for communicating important protocol modifications (eg, changes to eligibility criteria, outcomes, analyses) to relevant parties (eg, investigators, REC/IRBs, trial participants, trial registries, journals, regulators)
Consent or assent	26a	Who will obtain informed consent or assent from potential trial participants or authorised surrogates, and how (see Item 32)
	26b	Additional consent provisions for collection and use of participant data and biological specimens in ancillary studies, if applicable
Confidentiality	27	How personal information about potential and enrolled participants will be collected, shared, and maintained in order to protect confidentiality before, during, and after the trial
Declaration of interests	28	Financial and other competing interests for principal investigators for the overall trial and each study site
Access to data	29	Statement of who will have access to the final trial dataset, and disclosure of contractual agreements that limit such access for investigators
Ancillary and post-trial care	30	Provisions, if any, for ancillary and post-trial care, and for compensation to those who suffer harm from trial participation

For peer review only

For peer review only - http://bmjopen.bmj.com/site/about/guidelines.xhtml

Dissemination policy	31a	Plans for investigators and sponsor to communicate trial results to participants, healthcare professionals, the public, and other relevant groups (eg, via publication, reporting in results databases, or other data sharing arrangements), including any publication restrictions
	31b	Authorship eligibility guidelines and any intended use of professional writers
	31c	Plans, if any, for granting public access to the full protocol, participant-level dataset, and statistical code
**Appendices**		
Informed consent materials	32	Model consent form and other related documentation given to participants and authorised surrogates
Biological specimens	33	Plans for collection, laboratory evaluation, and storage of biological specimens for genetic or molecular analysis in the current trial and for future use in ancillary studies, if applicable

*It is strongly recommended that this checklist be read in conjunction with the SPIRIT 2013 Explanation & Elaboration for important clarification on the items. Amendments to the protocol should be tracked and dated. The SPIRIT checklist is copyrighted by the SPIRIT Group under the Creative Commons "Attribution-NonCommercial-NoDerivs 3.0 Unported" license.

For peer review only
